# Non-Invasive Skin Imaging Techniques for Diagnosing and Monitoring Mycosis Fungoides: A Comprehensive Review

**DOI:** 10.3390/cancers18182998

**Published:** 2026-09-16

**Authors:** Banu İsmail Mendi, Ceren İşibol, Alyssa Sayegh, Bökebatur Ahmet Raşit Mendi, Mehmet Fatih Atak, Banu Farabi

**Affiliations:** 1 Department of Dermatology, Faculty of Medicine, Ankara Yıldırım Beyazıt University, 06800 Ankara, Türkiye; 2 Department of Dermatology, Ankara Bilkent City Hospital, 06800 Ankara, Türkiye; ceren.isibol@saglik.gov.tr; 3 School of Medicine, New York Medical College, Valhalla, NY 10595, USA; asayegh4@student.nymc.edu; 4 Department of Radiology, Ankara Yıldırım Beyazıt University Faculty of Medicine, 06800 Ankara, Türkiye; barmendi@aybu.edu.tr; 5 Department of Dermatology, NYC Health + Hospitals/Metropolitan, New York, NY 10029, USA; atakm@nychhc.org; 6 Department of Dermatology, Icahn School of Medicine at Mount Sinai, New York, NY 10029, USA; banu.farabi@mountsinai.org

**Keywords:** mycosis fungoides, cutaneous T-cell lymphoma, non-invasive imaging, dermoscopy, optical coherence tomography

## Abstract

Mycosis fungoides, the most common primary cutaneous T-cell lymphoma, presents notable diagnostic challenges. In its early stages, it may resemble inflammatory dermatoses both clinically and histologically, causing delays in diagnosis. These factors can lead to treatment delays, allowing the disease to progress and adversely affecting the patient’s quality of life. To address this issue, the use of skin imaging techniques like dermoscopy, reflectance confocal microscopy, optical coherence tomography, and high-frequency ultrasound has increased in recent years. These techniques assist in diagnosis and treatment monitoring, reducing unnecessary biopsies by guiding accurate sampling from the most relevant sites and improving staging accuracy. This review provides a thorough overview of non-invasive skin imaging methods employed in the diagnosis and management of mycosis fungoides.

## Simple Summary

Mycosis fungoides, the most common type of cutaneous T-cell lymphoma, may be difficult to diagnose because it often resembles common inflammatory skin diseases. This may lead to delayed treatment and repeated skin biopsies. In recent years, non-invasive skin imaging techniques such as dermoscopy, reflectance confocal microscopy, optical coherence tomography, and high-frequency ultrasound have become increasingly useful in supporting diagnosis and follow-up. These methods can help clinicians identify suspicious lesions, select better biopsy sites, and monitor treatment response while reducing unnecessary invasive procedures. This review summarizes the current knowledge on non-invasive imaging findings in mycosis fungoides and highlights their potential role in improving patient care and guiding future research.

## 1. Introduction

Mycosis fungoides (MF) represents the most prevalent form of cutaneous T-cell lymphoma and often presents diagnostic challenges [1]. Diagnosing MF is especially difficult in the early stages, as both histopathological and clinical features may mimic inflammatory disorders. Consequently, diagnostic delays and the necessity for repeated biopsies are common occurrences. Therefore, choosing the right site for biopsy is crucial for an accurate diagnosis [2].

Although non-invasive imaging methods have not replaced traditional histopathology and immunophenotyping in diagnosing MF, they have proven useful as supplementary tools. These techniques help identify suitable lesions for biopsy, providing more precise disease staging and assessment of treatment response, while also potentially reducing unnecessary biopsies [3]. This review aims to explore the non-invasive imaging techniques that are used in diagnosing and monitoring MF.

## 2. Methods

### 2.1. Search Strategy/Eligibility Criteria

A comprehensive literature search was conducted using two online databases, PubMed and Embase, from database inception through April 2026. To ensure completeness, an additional targeted search was conducted on PubMed with broader criteria to identify studies that may not have been captured in the initial database queries. The comprehensive search strategy is detailed in Table 1.

To maintain a focused and comprehensive approach, our search strategy was limited to studies evaluating non-invasive skin imaging modalities in MF. Studies were eligible if they included human participants of any age or sex with a diagnosis of MF and investigated the use of imaging techniques, including high-frequency ultrasound (HFUS), reflectance confocal microscopy (RCM), dermoscopy, optical coherence tomography (OCT), line-field confocal OCT (LC-OCT), or trichoscopy. We included original research studies, including prospective and retrospective studies, cohort studies, case–control studies, cross-sectional studies, clinical trials, case series and reports, and diagnostic accuracy studies. Studies conducted in outpatient dermatology clinics, hospital-based dermatology units, tertiary referral centers, and specialty clinics were eligible, and no comparator was required for inclusion.

Exclusion criteria included animal studies, in vitro or ex vivo studies, review articles, systematic reviews, meta-analyses, editorials, expert opinions, and abstract-only conference publications without full data. Technical calibration studies and studies focused solely on algorithm development or image segmentation without clinical outcomes were also excluded. Only studies published in English, in peer-reviewed journals, and with full-text availability were included.

Included studies were grouped by imaging modality (dermoscopy/trichoscopy, RCM, OCT/LC-OCT, HFUS) and, when applicable, by MF subtype or clinical purpose (diagnosis, biopsy-site selection, monitoring).

### 2.2. Data Extraction

Covidence (Melbourne, Australia), a central online reference manager for systematic reviews, was used. Duplicates were removed, and two reviewers (AS and BARM) independently screened titles and abstracts for eligibility. Full-text articles were then reviewed by BİM and Cİ, and any discrepancies were resolved by consulting a third reviewer if needed. For each included study, the following data were extracted: author, year, sample size, patient demographics, and primary results.

### 2.3. Outcomes

The primary outcome was the assessment of the various clinical and diagnostic utilities of non-invasive imaging in MF.

### 2.4. Synthesis and Risk of Bias Assessment

Due to differences in methodology and data collection among the included studies, and the fact that the vast majority consisted of case series and case reports, an overall synthesis or risk-of-bias assessment was not possible. The included articles were grouped into five categories: dermoscopy, confocal microscopy, high-frequency ultrasound, LC-OCT, and other techniques. However, substantial heterogeneity was also observed within these categories, including variation in patient populations, such as the inclusion of different MF subtypes; differences in reported findings; and technical variation in imaging methods, including differences in frequency and light source. In addition, most studies included only a small number of patients.

## 3. Results

### 3.1. Literature Identification

**Figure 1:**
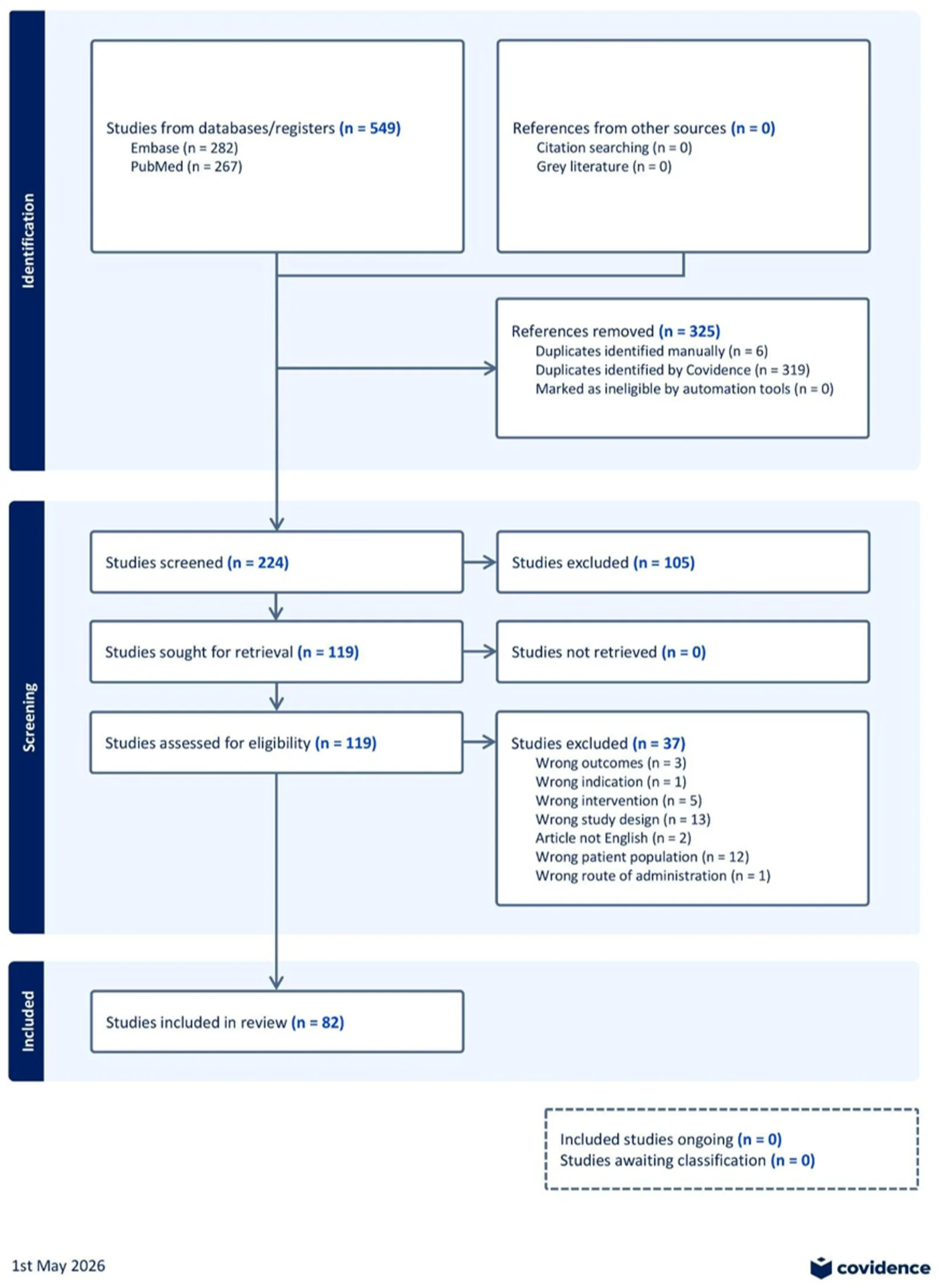
The PRISMA flow diagram of the selection process.

A total of 549 studies were imported for screening across both databases. After removing duplicates, 224 studies remained for title and abstract screening. Of these, 119 full-text studies were assessed for eligibility through full-text review. Ultimately, 82 studies met the inclusion criteria. The PRISMA flow diagram demonstrating the selection process is included in Figure 1.

### 3.2. Dermoscopy in Mycosis Fungoides

As a non-invasive, low-cost, and easily applicable diagnostic tool, dermoscopy is increasingly used in the diagnosis of MF, allowing visualization of vessel morphology and arrangement, scale patterns, and color. Many studies have been conducted in order to assess specific dermoscopic features of different stages of classical MF lesions, as well as other rarer subtypes. Those studies are important since dermoscopy can guide clinicians in differentiating between MF, otherwise known as the great mimicker, and other inflammatory dermatoses [4]. In our review, we found that 57 studies evaluated dermoscopy of MF skin lesions. Studies examining the dermoscopic features of MF skin lesions are summarized in Table 2.

In a study by Lallas et al. [5] dermoscopic features of 32 patients with patch-stage MF and 35 patients with chronic dermatitis were examined. Fine short linear vessels and orange-yellow patchy areas were the most common findings. A highly specific spermatozoa-like vascular pattern was observed only in MF and was absent in chronic dermatitis. White scales were occasionally present, whereas yellow scales were absent. Fine short linear vessels showed the highest sensitivity, while spermatozoa-like vessels and orange-yellow patchy areas demonstrated the highest specificity.

Balan et al. [6] evaluated 159 patients with early-stage MF, plaque psoriasis, or atopic dermatitis. Linear vessels were consistently observed in MF, together with spermatozoa-like vessels, focal white and orange structureless areas, a pink-to-pale background, and scaling along skin furrows. These features -predominant linear vessel type, scaling along skin furrows, and orange structureless areas- significantly distinguished MF from psoriasis and atopic dermatitis. Unlike Lallas et al. [5], Balan et al. found that fine linear and spermatozoa-like vessels may also occur in atopic dermatitis, suggesting reduced specificity in broader clinical settings.

Bilgiç et al. compared MF with a broader spectrum of inflammatory dermatoses and reported that light and dull red color, patchily arranged dotted and linear vessels, and patchy white scales were more prominent in the MF group compared to other dermatological diseases [7].

Chabbouh et al. [8] evaluated dermoscopic features of MF, plaque psoriasis, and chronic dermatitis in patients with Fitzpatrick skin phototypes III–V. Early-stage MF was characterized by polymorphic, serpentine, and linear vessels, predominantly arranged in an unspecific distribution, as well as reticular lines, an orange background, and structureless areas. Spermatozoa-like vessels, follicular plugs, comedo-like openings, spicules, dystrophic hairs, perifollicular hypo- or hyperpigmentation, circles, reticular lines, and perpendicular white lines were observed exclusively in MF.

Xu et al. [9] evaluated dermoscopic features of early-stage MF in a Chinese cohort. The most frequently observed dermoscopic features in MF were a dull red background, linear vessels, spermatozoa-like vessels, and orange-yellow patchy areas. In contrast to Chabbouh et al. [8], vascular distribution was predominantly regular. Linear vessels, spermatozoa-like vessels, and orange-yellow patchy areas demonstrated the highest diagnostic performance for differentiating MF from plaque psoriasis and contact dermatitis.

One study evaluated dermoscopic features of MF in 11 patients with skin of color (Fitzpatrick IV–VI). Because vascular structures are less readily visualized in darker skin, characteristic features included pseudonetworks of dark brown-to-gray or black clods and dots, erythematous-to-whitish pink structureless areas overlaid by thick black lines, prominent eccrine duct openings, white rosettes, and geometric white lines [10].

Errichetti et al. [11] evaluated dermoscopic features across different stages of classical MF and its variants. Patch-stage MF was characterized by linear vessels arranged in a reticular or unspecific pattern, with reticularly arranged linear vessels and white scales localized to skin furrows observed exclusively in this stage. Plaque-stage MF showed clustered dotted vessels as the only stage-associated vascular feature. In tumor-stage MF, peripheral linear vessels with branches were exclusive to this stage. Additional features included ulceration and red globules separated by white lines. Overall, the authors suggested that vascular morphology evolves with disease stage, with linear vessels predominating in patch-stage and branched linear vessels in tumor-stage lesions.

**Table 2:** Studies involving dermoscopic evaluation of classical MF skin lesions.

Studies	Type of Study	Type of Dermoscope	Patient Group	Findings (Number)
Errichetti et al. [11]	Cross-sectional Study (Multicenter)	NR	118 MF patients; 75 classical MF. (24 patch, 23 plaque, 28 tumoral), 26 folliculotropic, 9 erythrodermic, 8 poikilodermatous)	**Patch stage:** Dotted vessels: Uniform (5/24), unspecific (10/24); Linear vessels: reticular (3/24), unspecific (15/24); Linear curved vessels: uniform (1/24), unspecific (12/23); WS: patchy (15/24), in the skin furrows (5/24); patchy YSC (1/24); SA: focal white (bright) SA (5/24), focal orange SA (11/24), diffuse orange SA (1/24); purple dots/globules (3/24), unspecifically arranged (white bright) lines (1/24) **Plaque stage:** Dotted vessels: uniform (5/23), clustered (3/23), unspecific (11/23); unspecific linear vessels (8/23); unspecific linear curved vessels (1/23); WS: diffuse (2/23), peripheral (1/23), patchy (18/23); patchy YSC (5/23); follicular plugs (1/23); SA: focal white (bright) (14/23), focal brown SA (1/23), focal orange SA (7/23), diffuse orange SA (3/23); White (bright) lines: parallel (1/23), reticular (1/23), perpendicular (1/23), unspecifically arranged (3/23); brown lines (1/23), white circles (2/23) **Tumoral stage:** Dotted vessels: uniform (6/28), peripheral (3/28), unspecific (8/28); Linear vessels: peripheral (3/28), unspecific (8/28); Linear vessels with branches: uniform (1/28), peripheral (4/28), unspecific (3/28); Linear curved vessels: uniform (2/28), peripheral (2/28), unspecific (2/28); WS: diffuse (3/28), central (2/28), peripheral (4/28), patchy (11/28); YSC: peripheral (1/28), patchy (2/28); follicular plugs (2/28); SA: focal white (bright) (16/28), focal grey (1/28), focal orange (14/28), diffuse orange (3/28), focal purple (1/28); white dots (4/28); white globules (2/28); orange globules (2/28); purple dots (1/28); purple globules (2/28); White (bright) lines: parallel (1/28), reticular (2/28), perpendicular (1/28), unspecifically arranged (3/28); white circles (5/28); ulceration (6/28); red globules separated by white lines (4/28)
Bilgiç et al. [7]	Case–Control Study	Dermlite DL200HR, ×10 magnification	150 patients; 50 plaque psoriasis, 30 lichen planus, 20 MF, 30 pityriasis rosea, 20 nummular dermatitis	Background color: Light red (9/20), dull red (2/20), yellow (2/20), yellow-orange (7/20); dotted vessels (7/20), dotted+linear vessels (13/20); Vessel arrangement: regular (3/20), clustered (1/20), patchy (16/20); Scale color: white (16/20), yellow-white (1/20); Scale distribution: patchy (11/20), peripheral (1/20), diffuse (5/20)
Nakamura et al. [10]	Cross-sectional study	NR	11 patients with skin of color (Fitzpatrick IV–VI); 4 classic, 4 hypopigmented, 1 folliculotropic, 2 verrucous	Formation of pseudonetworks made up of dark brown to gray or black clods and dots; thick black lines overlying erythematous to whitish pink structureless zones interrupted by prominent eccrine duct openings; white rosettes; geometric white lines
Soliman et al. [12]	Cross-sectional study	Dermlite II PRO HR (3Gen) using polarized light, magnification ×10	88 patients; 32 patch stage classical, 16 with both patch and tumor stage classical, 20 hypopigmented, 8 poikilodermatous, 4 folliculotropic, 4 erythrodermic, 4 hyperpigmented	**Patch stage:** Background: non-homogenous (32/32), pink to erythematous (32/32); patchy whitish scales (28/32); patchy areas of orange discoloration (28/32); Globules: blue gray (12/32), dark (12/32), patchy red (4/32), pearly white (4/32); dotted blood vessels (28/32); short linear blood vessels (28/32); spermatozoa-like blood vessels (16/32) **Patch and tumor stage:** Background: non-homogenous (16/16), pink to erythematous (16/16); patchy whitish scales (16/16), patchy areas of orange discoloration (16/16); Globules: blue gray (12/16), dark (12/16), patchy red (4/16); dotted blood vessels (16/16); short linear blood vessels (16/16); spermatozoa-like blood vessels (16/16)
Ali et al. [1]	Prospective cross-sectional study	Dermlite DL4,3 Gen, Inc, San Juan Capist- rano, CA, USA with ×10 magnification	53 patients; 10 patch stage classical, 9 plaque stage classical, 3 tumor stage classical, 16 hypopigmented, 7 hyperpigmented, 5 poikilodermatous, 3 erythrodermic	**Patch stage:** Vascular changes: fine linear short vessels (7/10), dotted vessels (4/10), spermatozoa-like vessels (6/10), thick linear vessels (3/10), polymorphous pattern of vessels (5/10); Scales: white (10/10), geometric white (10/10), perifollicular white (5/10), lamellar (3/10); orange yellowish patches (9/10); white structureless patches (6/10); brownish pigmentation (3/10); follicular plugging (1/10) **Plaque stage:** Vascular changes: fine linear short vessels (7/9), dotted vessels (4/9), spermatozoa-like vessels (4/9), polymorphous pattern of vessels (4/9); Scales: white (9/9), geometric white (8/9), perifollicular white (6/9), lamellar (3/9); orange yellowish patches (7/9); white structureless patches (4/9); whitish pink structureless patches (4/9); brownish pigmentation (6/9); perifollicular and perieccrine duct openings hyperpigmented halo (2/9); prominent eccrine duct openings (4/9) **Tumor stage:** Vascular changes: fine linear short vessels (3/3), dotted vessels (2/3), polymorphous pattern of vessels (1/3), purpuric spots (2/3); Scales: white (3/3), geometric white (3/3), perifollicular white (2/3), lamellar (1/3); orange yellowish patches (3/3), white structureless patches (3/3); whitish pink structureless patches (3/3); ulceration (1/3)
Lallas et al. [5]	Case–Control Study	Ecoscan II V0990.21, Canon 1000D, original magnification ×10	67 patients; 32 patch stage classical MF, 35 CD	Vessels: Fine short linear (30/32), dotted (18/32), spermatozoa-like (16/32 in MF, 0 in CD); orange-yellowish patchy areas (29/32 in MF, 0 in CD); white scales (6/32); yellow scales (0 in MF, 20/35 in CD)
Chabbouh et al. [8]	Multicenter cross-sectional study	DermLite DL4^©^, 3GEN, US	89 patients with Fitzpatrick III-V; 34 early-stage MF, 37 plaque psoriasis, 18 CD	Vessel type: dots (29/34), linear (26/34), spermatozoa-like (41%), polymorphic (28/34), monomorphous (6/34), serpentine (14/34), coiled (7/34); Vessel arrangement: nonspecific (32/34), branched (2/34); white scale (33/34); yellow scale (1/34); diffuse scale (1/34); patchy scale (33/34); linear scale (3/34); Structureless area color: orange (2/34), white (8/34); Background color: red (1/34), orange (15/34), yellow (1/34), pink (23/34); follicular spicules (6/34); follicular plugs (5/34); perifollicular white color (3/34); perifollicular pigmentation (1/34); comedo-like openings (3/34), dystrophic hairs (3/34); Lines: reticular (16/34), perpendicular white (1/34); Clods color: white (7/34), yellow-orange (10/34), gray (1/34); circle (2/34)
Xu et al. [9]	Case–control Study	Medicam 800HD, FotoFinder Systems GmbH, Birbach, Germany) at 20-fold magnification.	101 patients; 31 early MF, 36 CD, 34 plaque psoriasis	Regularly distributed linear vessels and spermatozoa-like structures on a dull-red background; orange-yellowish areas
Balan et al. [6]	Prospective case–control study	DermLite DL5	159 patients; 55 early MF, 53 psoriasis, 51 atopic dermatitis	Linear vessels (55/55); spermatozoa-like vessels (51/55); dotted vessels (53/55); Vascular pattern: clustered (18/55), nonspecific (37/55); Predominant vessel type: linear (53/55), dotted (2/55); polymorphous vessel pattern (53/55); presence of scale (51/55); presence of white scale (51/55); White scale distribution: along skin furrows (34/55), patchy (15/55), peripheral (2/55); hemorrhagic globules (3/55); focal white structureless areas (48/55); focal orange structureless areas (43/55); ulceration (4/55); dystrophic hair (34/55); Background color: pink or pale red (49/55), orange (5/55), bright red (1/55)
Zychowska et al. [13]	Case–Control Study	Canfield D200	62 patients; 7 MF, 12 dermatomyositis, 19 psoriasis, 16 chronic dermatitis, 5 lichen planus, 3 pityriasis rubra pilaris	Vessel morphology: dotted (7/7), linear (1/7), linear curved (1/7), pleomorphic (1/7); Vessel distribution: uniform (3/7), clustered (3/7), unspecified (3/7), ring pattern (1/7); Scales: white (5/7), yellow (2/7); Scale distribution: diffuse (2/7), patchy (3/7); follicular plugs (1/7); perifollicular white color (1/7); Structureless areas: white (2/7), pink (1/7), yellow (1/7); red dots and globules (2/7), perivascular white halo (3/7)
Bosseila et al. [14]	Prospective case–control study	Dermlite II Hybrid with a magnification of ×10 (3Gen, USA)	25 patients; 16 classical, 7 hypopigmented, 2 poikilodermatous	Dotted and linear vessels
Öztürk et al. [15]	Prospective case–control study	Dermlite DL4 PigmentBoost Plus X10; 3 Gen, San Juan Capistrano, CA, USA	34 patients; 17 with stage IIA MF, 17 with plaque psoriasis	Orange-yellow patches (15/17); fine, short, linear vessels (14/17); geometric white scales (12/17); perifollicular white scales (8/17); purpuric dots (6/17); collarette white scales (6/17); white patches (6/17); spermatozoa-like structures (5/17); Y shaped arborizing vessels (5/17); Dotted vessels (5/17); Diffuse lamellar white scales (1/17); Dotted and globular vessels (1/17)
Erdil et al. [16]	Prospective, observational, cross-sectional study	FotoFinder Systems GmbH	59 patients; 28 early-stage MF, 31 small plaque psoriasis	Short linear vessels (24/28); linear curved vessels (15/28); dotted vessels (19/28); branching linear vessels (11/28); Vascularity pattern: none (2/28), peripheral (1/28), uniform (6/28), clustered (1/28), nonspecific (18/28); purpuric area (7/28); scale (11/28); Localization of scale: patchy (6/28), diffuse (5/28); follicular findings (4/28); dilated follicle (2/28); structureless orange area (22/28); Structureless orange area feature: patchy (23/28), diffuse (1/28); structureless white area (9/28); purple color (8/28); brown reticular network/poikiloderma (3/28)
Ghahramani et al. [17]	Prospective cross-sectional study	NR	15 patients; 7 MF (6 patch, 1 plaque), 5 FMF, 1 lymphomatoid papulosis, 2 cutaneous B-cell lymphoma	**Patch stage:** Structureless patches (6/6), fine short linear vessels (5/6), dotted vessels (1/6), spermatozoa-like structures (4/6), white scale (3/6), light red background (1/6), dull red background (3/6), crystalline structures (2/6) **Plaque stage:** Structureless patches (1/1), dotted vessels (1/1), orange-yellowish patchy areas (1/1), white scale (1/1), dull red background (1/1)
Jasińska et al. [18]	Case–Control study	Fotofinder digital dermoscope (20–70× magnification)	55 patients; 21 MF, 21 psoriasis, 13 eczematous dermatitis	Lack of hair (6/21); Hair shafts: pili torti (12/15) (numerous pili torti (8/12), single pili torti (4/12)), 8 shaped hairs (3/15), pigtail hairs (2/15), broken hairs (8/15); hair shafts rapidly tapered over long sections (7/15); angulated hairs (8/15); branched hairs (2/15); Follicular ostia: yellow dots (17/21), black dots (1/21)
Zychowska et al. [19]	Prospective case–control study	Canfield D200^EVO^ Video- dermatoscope (15–30-fold magnification). For better evaluation of scaling, examination without immersion fluid was performed as the first step. Then, “wet dermoscopy” (with ultrasound gel) was performed for the assessment of capillaries and other structures	139 patients with erythematosquamous skin diseases (27 SCLE, 36 psoriasis, 30 nummular dermatitis, 26 MF, 20 pityriasis rosea	Vessel morphology: dotted (23/26), linear (10/26), linear with branches (4/26), thick (1/26), thin (4/26), linear curved (9/26), polymorphous (14/26); Distribution of vessels: peripheral (2/26), unspecific (22/26); Color of scales: white (19/26), yellow (3/26); Distribution of scales: diffuse (7/26), peripheral (5/26), patchy (12/26), along skin lines (1/26); Follicular findings: follicular plugs (2/26), perifollicular white halo (2/26), perifollicular scaling (1/26); Structureless areas: white (4/26), pink (6/26), yellow (5/26); dots/globules (14/26); red dots (9/26); red globules (7/26); gray-brown dots (4/26); gray-brown globules (1/26); white lines (1/26); circles (1/26); dull pink background (15/26); pink-red background (11/26); red hemorrhagic areas (6/26); pigmentation (9/26); peripheral pigmentation (1/26); yellow-orange round structures (1/26)
Zengarini et al. [4]	Cross-sectional study	NR	22 patients with cMF, 4 patients with FMF, and 4 patients with SS	Linear vessels (12/30), serpentine vessels (4/30), dotted vessels (11/30). 12/30 lesions showed a diffuse vessel distribution, and 11/30 showed perifollicular vessel distribution. Background color: Red in 17/30 cases, orange in 12/30 cases, and brown in 1/30 case. Diffuse scaling in 12/30 cases, perifollicular scaling in 11/30 cases. Keratin plugs were present in 12/30 cases, and they were predominantly yellow.
Wohlmuth-Wieser et al. [20]	Case–control study	DermLite DL4^®^ (3Gen Inc., San Juan Capistrano, CA, USA) with 10-fold magnification	13 patients; Six of the patients had CTCL (2 patch stage MF, 1 plaque stage MF, 1 FMF, 2 SS), 5 had psoriasis, and 2 had AD	**Patch stage:** Fine short linear vessels (2/4), dotted vessels (2/4), spermatozoa-like structures (2/4), light red background (3/4), dull red background (1/4), white scale (3/4), structureless patches (2/4), crystalline structures (1/4) **Plaque stage:** Dotted vessels (1/1), dull red background (1/1), white scale (1/1) **FMF:** Fine short linear vessels (1/1), dotted vessels (1/1), perifollicular accentuation (1/1), comedo-like openings (1/1)
Tkach et al. [21]	Case report	NR	A case of transformation of psoriasis into MF in a 52-year-old male patient	White circles and lumps against the background of small-textured pink-red areas with single dots, looped and spermatozoa-like blood vessels
Belcadi et al. [22]	Case report	NR	MF mimicking a nodule of Sister Mary Joseph in a 45-year-old female patient	Nonmelanocytic lesion with comedo-like openings

**Abbreviations:** MF, mycosis fungoides; NR, not reported; YSC, yellow scales/crusts; WS, white scales; SA, structureless areas; CD, chronic dermatitis; SCLE, subacute cutaneous lupus erythematosus; cMF, classic mycosis fungoides; FMF, folliculotropic mycosis fungoides; SS, Sezary syndrome; LPP, lichen planopilaris.

#### 3.2.1. Folliculotropic Mycosis Fungoides

Twenty-one studies were involved in the evaluation of dermoscopic features of folliculotropic MF lesions (Table 3). Despite the lack of extensive studies, there are a number of dermoscopic features described in FMF, such as perifollicular accentuation, which is a white halo around the follicle, comedo-like openings, white structureless areas, and dotted or fine linear vessels [23]. Accentuation of hair follicles in patients with MF might be an early sign of folliculotropism, which can be an important clue for detecting FMF in the setting of a subtle clinical presentation [24].

Errichetti et al. [11] evaluated 26 FMF patients. The most common findings were follicular plugs, dilated follicles, and alopecia. Gallo et al. [25] reported non-scarring alopecia features in over half of their FMF cohort, including a decreased number of pilosebaceous units, dotted dilated vessels, broken and dystrophic hairs, vellus hairs, spermatozoa-like vessels, and yellow dots. Features associated with both scarring and non-scarring alopecias were present in a minority of their cohort. Short hairs with split ends were exclusive to patchy-plaque alopecia, whereas pigtail hairs were absent in plaque-stage but present in 50% of patchy-stage disease. Pigtail hairs, yellow dots, and spermatozoa-like vessels predominated in early-stage disease, confirming pigtail hairs as an early marker. Soliman et al. [12] identified non-homogenous pink-to-erythematous backgrounds, patchy scales, perifollicular and interfollicular scaling, orange discoloration, blue gray globules, dark globules, and varied vascular patterns (dotted, short linear, spermatozoa-like, arborizing). Comedo-like openings were highlighted as a distinctive feature of FMF compared to other MF subtypes.

#### 3.2.2. Poikilodermatous Mycosis Fungoides

There were 8 studies evaluating the dermoscopic features of poikilodermatous MF lesions. Soliman et al. [12] examined 8 patients with PMF in their study. Key observations included a non-homogenous pink-to-erythematous background with patchy areas of orange discoloration, along with dotted, short linear, spermatozoa-like, and arborizing vessels.

Ali et al. [1] evaluated 53 patients, 5 of whom presented with PMF. White scales, white structureless patches, whitish pink structureless areas, and brownish pigmentation were present in all cases. Other features were fine linear short vessels, spermatozoa-like vessels, thick linear vessels, polymorphous pattern of vessels, dotted vessels, purpuric spots, and geometric white scales.

Errichetti et al. [11] included 8 patients with PMF. The most indicative features were focal white and brown structureless areas (checkerboard pattern), brown reticular lines, and patchy white scales, although vascular structures were less visible.

#### 3.2.3. Hyperpigmented Mycosis Fungoides

There were 3 studies evaluating dermoscopic features of hyperpigmented MF lesions. In the study by Soliman et al. [12], 4 individuals presented with the hyperpigmented variant of the disease. All 4 patients demonstrated a non-homogeneous pink-to-erythematous background, patchy white scales, patchy orange discoloration, blue-gray and dark globules, multifocal pigmentation, dotted vessels, and short linear vessels. Among all evaluated subtypes, multifocal pigmentation was a feature unique to the hyperpigmented variant.

Ali et al. [1] evaluated 53 patients, 7 of whom had hyperpigmented MF. Characteristic findings included white scales, geometric white scales, brownish pigmentation, fine short linear vessels, perifollicular white scales, orange-yellowish patches, and prominent eccrine duct openings. Less common findings were dotted vessels, polymorphous vessels, hyperpigmented perifollicular/perieccrine halos, spermatozoa-like vessels, and follicular pigmentation. Unlike other variants studied, follicular pigmentation was seen exclusively in the hyperpigmented variant.

#### 3.2.4. Hypopigmented Mycosis Fungoides

Four studies evaluated the lesions of hypopigmented MF dermoscopically. In a small cohort of patients with skin of color [10], dermoscopic features were patchy, amorphous, white-pink areas with reduction or loss of the patients’ natural pigment network.

In a larger cohort [12], 20 patients had hypopigmented MF. Foggy whitish areas and hypopigmented areas were seen in 100% of the patients. Other features were pink-to-erythematous background (20%), patchy areas of orange discoloration (20%), and pearly white globules (60%). Unlike the other MF variants evaluated, foggy whitish areas and hypopigmented areas were observed exclusively in the hypopigmented subtype.

Ali et al. [1] evaluated 16 patients with the hypopigmented variant. Thick linear blood vessels (42.9%), white structureless patches (100%), whitish pink structureless areas (100%), and disturbed pigment network (100%) were observed. Among these, a disturbed pigment network was a distinctive feature of the hypopigmented variant that was not seen in other included variants (classic, hypopigmented, poikilodermatous, erythrodermic).

#### 3.2.5. Erythrodermic Mycosis Fungoides

Eight studies evaluated the dermoscopic and trichoscopic features of erythrodermic MF lesions (EMF), five of which focused on differentiating EMF from other causes of erythroderma. Sławinska et al. [26] analyzed 28 erythrodermic patients, including 9 with erythrodermic MF. All EMF patients demonstrated dotted and linear vessels, unspecific vessel distribution, white scale color, patchy scale distribution, and white-pinkish structureless areas on dermoscopy. Pink structureless areas, perifollicular pigmentation on trichoscopy, and gray dots on trichoscopy were unique to MF. Other dermoscopic findings included branched linear vessels, linear curved vessels, and orange or brown structureless areas. Additional features comprised dotted or linear vessels (nonspecific distribution), white or yellow interfollicular scales, interfollicular pigmentation, and red, pink, or white-pinkish structureless areas.

Golinska et al. [27] evaluated 49 patients with erythroderma including 7 with EMF. Interfollicular white scales, irregular vessel distribution, and whitish-pinkish structureless areas were observed in all EMF patients. Yellow scales, dotted vessels, short linear irregular vessels, spermatozoon-like vessels, glomerular vessels, branched vessels, serpentine vessels, orange structureless areas, interfollicular pigmentation, and perifollicular pigmentation were also common. Spermatozoa-like vessels were exclusive to cutaneous T-cell lymphoma, including both EMF and Sézary syndrome.

Rakowska et al. [28] studied trichoscopic features in 56 patients with erythroderma. High-frequency findings included white interfollicular scales, yellow dots, numerous pili torti, numerous broken hairs, and reddish areas. Yellow dots and white interfollicular scales yielded the highest diagnostic sensitivity, while numerous pili torti and yellow dots showed the highest specificity. Pili torti, anagen bulbs, 8-shaped hairs, black dots, thick white interfollicular bands, patchy hyperpigmentation, and spicule-like scales were seen exclusively in the CTCL group.

Errichetti et al. [11] evaluated 9 EMF patients among a total of 118 MF cases. The most frequent features were dotted vessels, linear curved vessels, white patchy scales, focal orange structureless areas, focal brown structureless areas, focal white structureless areas, and diffuse orange structureless areas.

**Table 3:** Studies involving dermoscopic evaluation of MF variants.

Mycosis Fungoides Variant	Studies	Type of Study	Type of Dermoscope	Patient Group	Findings (Number)
**Folliculotropic MF**	Errichetti et al. [11]	Cross Sectional Study (Multicenter)	NR	118 MF patients (75 classic, 26 folliculotropic, 9 erythrodermic, 8 poikilodermatous)	Dotted vessels: Unspecific (5/26), peripheral (2/26), uniform (1/26), clustered (1/26); Linear curved vessels: unspecific (2/26), peripheral (2/26); Linear vessels with branches: unspecific (2/26), peripheral (1/26); White scales: patchy (9/26), diffuse (2/26); yellow scales and crusts (1/26); follicular plugs (16/26), follicular red dots (8/26), perifollicular white color (8/26); focal white structureless areas (3/26), white circles (2/26); dilated follicles (21/26); lack of hairs (24/26)
	Nakamura et al. [10]	Cross sectional study	NR	11 patients with skin of color (Fitzpatrick IV–VI); 4 classic, 4 hypopigmented, 1 folliculotropic, 2 verrucous	Follicular plugging, perifollicular hyperpigmentation
	Soliman et al. [12]	Cross-sectional study	Dermlite II PRO HR (3Gen) using polarized light, magnification ×10	88 patients; 32 patch stage classical, 16 with both patch and tumor stage classical, 20 hypopigmented, 8 poikilodermatous, 4 folliculotropic, 4 erythrodermic, 4 hyperpigmented	Non-homogenous background (4/4), patchy white scales (4/4), perifollicular and interfollicular scaling (4/4), pink to erythematous background (4/4), patchy areas of orange discoloration (4/4), blue gray globules (4/4), dark globules (4/4), comedo-like openings (4/4), dotted blood vessels (4/4), short linear blood vessels (4/4), spermatozoa-like vessels (4/4), arborizing blood vessels (4/4)
	Gallo et al. [25]	Cross-sectional study	FotoFinder Dermoscope (TeachScreen Software GmbH, Bad Birnbach, Germany)	18 patients with FMF (9 at stage Ia, 3 at stage Ib, 4 at stage Iib, 2 at stage IIIa)	**Early FMF (IA-IB):** Single hairs (11/12), dotted dilated vessels (10/12), dystrophic hairs (6/12), vellus hairs (6/12), spermatozoa-like vessels (8/12), yellow dots (8/12), dilation of follicular openings (6/12), scales-crusts (4/12), white dots/lines (2/12), short hair with split end (2/12), pigtail hair (5/12), purpuric dots (3/12), absence of follicular dots (1/12), perifollicular hyperkeratosis (3/12), black dots (1/12), milky-white globules (1/12) **Advanced FMF (IIB-III):** Single hairs (5/6), dotted dilated vessels (4/6), dystrophic hairs (6/6), vellus hairs (4/6), spermatozoa-like vessels (2/6), yellow dots (2/6), dilation of follicular openings (2/6), scales-crusts (4/6), white dots/lines (4/6), short hair with split end (3/6), purpuric dots (2/6), absence of follicular dots (3/6), perifollicular hyperkeratosis (1/6), black dots (2/6), milky-white globules (2/6)
	Wang et al. [24]	Case series	NR	Patch stage MF patients with folliculotropism in histopathology	Yellow globules with sparsely distributed fine, short, linear vessels in the periphery and loss of normal hair-follicle architecture, perifollicular accentuation
	Tončić et al. [29]	Case report	NR	2 patients with syringotropic MF	Obliteration of the follicles, follicular accentuation, follicular plugging, loss of terminal follicles, comedo-like openings, interconnected regular-appearing structureless patches, bluish structures with unsharp borders
	Damiani et al. [30]	Case report	NR	10-year-old boy with FMF	Perifollicular hyperkeratosis with patchy background erythema, broken hairs at various lengths, hair casts, scattered perifollicular pustules, irregularly distributed horn plugs, and white halos. Some of the findings are commonly visible features of inflammatory alopecias but some differences were defined; background erythema was found to be perifollicular and sharply demarcated and hair casts were associated with perifollicular scaling rather than perifollicular erythema unlike LPP, horn plugs were irregularly distributed in contrast to those of DLE, perifollicular pustules were scattered unlike confluent pustules of FD. The authors suggested that perifollicular hyperkeratosis, broken hairs at various lengths, and irregularly distributed white halos could serve as early clues to FMF.
	Geller et al. [23]	Case series	NR	4 patients with FMF or follicular lymphoproliferative disorder	Orange-pink perifollicular clods with peripheral scale and central broken hairs; perifollicular halos; short fine vessels and perifollicular scale overlying a yellowish background, surrounding central keratotic plugs
	Ghahramani et al. [17]	Prospective cross-sectional study	NR	15 patients; 7 MF (6 patch, 1 plaque), 5 folliculotropic MF, 1 lymphomatoid papulosis, 2 cutaneous B-cell lymphoma	Structureless patches (3/5), perifollicular accentuation (5/5), comedo-like openings (3/5), fine short linear vessels (2/5), dotted vessels (2/5), spermatozoa-like structures (1/5), white scale (4/5), light red background (1/5), dull red background (4/5), crystalline structures (2/5), yellow ulceration (1/5)
	Caccavale et al. [31]	Case report	NR	59-year-old male patient with FMF	Perifollicular erythema surrounding comedo-like lesions, white structureless areas replacing lost hair follicles, and fine, short, linear, glomerular, dotted vessels
	Santos-Coelho et al. [32]	Case report	NR	50-year-old male patient with FMF	Decreased number of pilosebaceous units, dilated follicular openings (some with milky-white globules) with perifollicular accentuation, black dots, vellus and dystrophic hairs, widespread white scale, dotted vessels, spermatozoa-like vessels
	Kituashvili et al. [33]	Case report	NR	39-year-old male patient with FMF	Linear vessels, perifollicular scaling with erythematous background, white structureless area, dotted vessels
	Kreutzer et al. [34]	Case report	NR	59-year-old male patient with FMF	Scarring alopecia with loss of adnexal structures
	Billah et al. [35]	Case report	NR	11-year-old boy with FMF	Follicular plugs, peripilar scales with broken hairs, follicular plugs, structureless white-yellowish areas replacing lost hair follicles, short and dotted vessels on erythematous background
	Rodrigues et al. [36]	Case report	NR	A 42-year-old male patient presenting with milia en plaque associated with FMF.	Pale, sclerotic regions resulting from fibrosis due to follicular damage around yellowish papules resembling milia, visible blood vessels
	Aldayhum et al. [37]	Case report	NR	52-year-old male patient with FMF	Areas of hypopigmentation, hyperpigmentation, telangiectasia, and atrophy associated with terminal hair thinning
	Takahagi et al. [38]	Case report	NR	60-year-old male patient presenting with normolipemic xanthoma associated with FMF	Scaling and crusting at the hair shafts and follicles, and milky white structures in the perifollicular region, dilated follicles lacking hair surrounded by yellowish white structures
	Sławinska et al. [39]	Case report	NR	88-year-old female patient with FMF	Decreased number of pilosebaceous units within patchy areas of hair loss, single hairs, milky-white globules, yellow dots with or without centrally located black dots/broken hairs, short hair with split-end, short hair with triangular-shape end, short, broken hair, pigtail appearance hair and short hairs broken at the same or different level, white dots and lines replacing hair follicles, white and yellow scale
	Miteva et al. [40]	Case series	NR	2 patients with alopecia universalis associated with FMF	Patient 1: Diffuse erythema and empty follicular openings filled with keratotic plugs or filiform spicules. Patient 2: Diffuse erythema with mild scale, black dots, and sparse broken hairs
	Asz-Sigall et al. [41]	Case report	NR	58-year-old female patient with scalp FMF presenting as LPP	Reduced hair density (1–2 hairs per follicular unit), white structureless areas, fine spermatozoa-like blood vessels, dystrophic hairs, perifollicular erythema, and scaling
	Montoya et al. [42]	Case report	NR	64-year-old female patient with FMF presenting progressive diffuse alopecia, scalp nodules, leonine facies, and multiple erythematous plaques	Empty follicular openings, 3D yellow dots, follicular clefts with emerging tufts of hair, broken hairs, polytrichia, structureless yellow areas, erythematous-violaceous areas, and yellowish-brown dots with a whitish halo
**Poikilodermatous MF**	Errichetti et al. [11]	Cross Sectional Study (Multicenter)	NR	118 MF patients (75 classic, 26 folliculotropic, 9 erythrodermic, 8 poikilodermatous)	Dotted vessels: unspecific (2/8), uniform (1/8); Linear vessels: (2/8), reticular (1/8); white patchy scales (5/8); focal white structureless areas (7/8); focal brown structureless areas (4/8); focal orange structureless areas (3/8); diffuse orange structureless areas (1/8); focal purple structureless areas (1/8); brown reticular lines (4/8); white perpendicular lines (1/8); purple dots (2/8); white dots (1/8); white globules (1/8)
	Soliman et al. [12]	Cross-sectional study	Dermlite II PRO HR (3Gen) using polarized light, magnification ×10	88 patients; 32 patch stage classical, 16 with both patch and tumor stage classical, 20 hypopigmented, 8 poikilodermatous, 4 folliculotropic, 4 erythrodermic, 4 hyperpigmented	Non-homogeneous background (8/8), pink to erythematous background (8/8), patchy areas of orange discoloration (8/8), dotted vessels (8/8), short linear vessels (4/8), spermatozoa-like vessels (4/8), arborizing vessels (8/8)
	Ali et al. [1]	Prospective cross-sectional study	Dermlite DL4,3 Gen, Inc, San Juan Capist- rano, CA, USA with ×10 magnification	53 patients; 10 patch stage classical, 9 plaque stage classical, 3 tumor stage classical, 16 hypopigmented, 7 hyperpigmented, 5 poikilodermatous, 3 erythrodermic	Vascular changes: Fine linear short vessels (4/5), dotted vessels (1/5), spermatozoa-like vessels (4/5), thick linear vessels (3/5), polymorphous pattern of vessels (2/5), purpuric spots (3/5); Scales: white (5/5), geometric white (3/5); Color changes: white structureless patches (5/5), whitish pink structureless areas (5/5), brownish pigmentation (5/5)
	Nojima et al. [43]	Case report	NR	38-year-old female patient with CD8+ and CD56+ cytotoxic variant of poikilodermatous MF	Reticular brown areas with fine grey dots, dotted and glomerular vessels on a pinkish-white background
	Apalla et al. [44]	Case report	NR	68-year-old female patient with poikilodermic MF	Linear branching vessels arranged as a network in a pale pink-brown background, with fine, sparse white scales
	Xu et al. [45]	Case report	NR	59-year-old male patient with poikilodermic MF	Multiple polygonal structures consisting of lobules of white storiform streaks, studded with fine red dots or hairpin vessels; between the lobules were septa of pigmented dots that were unevenly and intermittently distributed throughout; red and yellowish smudges
	Sinha et al. [46]	Case report	NR	56-year-old male patient	Reticular brown pigment pattern on a pink-white background, with unevenly distributed grey dots present throughout the pigment pattern
	Bosseila et al. [14]	Prospective case–control study	Dermlite II Hybrid with a magnification of ×10 (3Gen, USA)	25 patients; 16 classical, 7 hypopigmented, 2 poikilodermatous	Light brown focal hyperpigmentation, arborizing blood vessels
**Hypopigmented MF**	Nakamura et al. [10]	Cross-sectional study	NR	11 patients with skin of color (Fitzpatrick IV–VI); 4 classic, 4 hypopigmented, 1 folliculotropic, 2 verrucous	Patchy, amorphous, white-pink areas with reduction or loss of the patient’s natural pigment network
	Soliman et al. [12].	Cross-sectional study	Dermlite II PRO HR (3Gen) using polarized light, magnification ×10	88 patients; 32 patch stage classical, 16 with both patch and tumor stage classical, 20 hypopigmented, 8 poikilodermatous, 4 folliculotropic, 4 erythrodermic, 4 hyperpigmented	Pink to erythematous background (4/20), patchy areas of orange discoloration (4/20), foggy whitish areas (20/20), hypopigmented areas (20/20), pearly white globules (12/20)
	Ali et al. [1]	Prospective cross-sectional study	Dermlite DL4,3 Gen, Inc, San Juan Capist- rano, CA, USA with ×10 magnification	53 patients; 10 patch stage classical, 9 plaque stage classical, 3 tumor stage classical, 16 hypopigmented, 7 hyperpigmented, 5 poikilodermatous, 3 erythrodermic	Thick linear vessels (6/16), white structureless patches (16/16), whitish pink structureless areas (16/16), disturbed pigment network (16/16)
	Soliman et al. [47]	Observational cross-sectional analytical study	Dermlite 4 (4Gen, San Juan Capistrano, CA, USA)	168 patients (38 vitiligo, 36 depigmented nevus, 16 hypopigmented MF, 16 idiopathic guttate hypomelanosis, 15 pityriasis alba, 17 hypopigmented pityriasis versicolor, 15 lichen sclerosis et atrophicus, 15 ash macule leaf of tuberous sclerosis)	Patterns of pigmentation: white structureless area (1/16), nebulous pattern (1/16); Pigment network: faint pigment network (14/16), reduced pigment network (2/16); Border: well defined border (2/16), ill-defined border (14/16); leukotrichia (1/16); vascular structures (6/16)
**Hyperpigmented MF**	Soliman et al. [12]	Cross-sectional study	Dermlite II PRO HR (3Gen) using polarized light, magnification ×10	88 patients; 32 patch stage classical, 16 with both patch and tumor stage classical, 20 hypopigmented, 8 poikilodermatous, 4 folliculotropic, 4 erythrodermic, 4 hyperpigmented	Non-homogenous background (4/4), patchy whitish scales (4/4), pink to erythematous background (4/4), patchy areas of orange discoloration (4/4), blue gray globules (4/4), dark globules (4/4), multifocal pigmentation (4/4), dotted blood vessels (4/4), short linear blood vessels (4/4),
	Ali et al. [1]	Prospective cross-sectional study	Dermlite DL4,3 Gen, Inc, San Juan Capist- rano, CA, USA with ×10 magnification	53 patients; 10 patch stage classical, 9 plaque stage classical, 3 tumor stage classical, 16 hypopigmented, 7 hyperpigmented, 5 poikilodermatous, 3 erythrodermic	Vascular changes: fine linear short vessels (5/7), dotted vessels (3/7), spermatozoa-like vessels (2/7), polymorphous pattern (3/7); Scales: white scales (7/7), geometric white scales (7/7), perifollicular white scales (5/7); Color changes: orange yellowish patches (4/7), brownish pigmentation (6/7), perifollicular and perieccrine duct openings hyperpigmented halo (3/7), follicular pigmentation (2/7); prominent eccrine duct openings (4/7)
	Zha et al. [48]	Case report	NR	A 52-year-old male patient developed a hyperpigmented MF in a lichen planus pigmentosus lesion	Indistinct white reticular streaks scattered among blue-gray dots with follicular plugs
**Erythrodermic MF**	Errichetti et al. [11]	Cross Sectional Study (Multicenter)	NR	118 MF patients (75 classic, 26 folliculotropic, 9 erythrodermic, 8 poikilodermatous)	Dotted vessels: unspecific distribution (4/9), peripheral distribution (2/9), clustered distribution (1/9); Linear vessels: reticular distribution (4/9), unspecific distribution (3/9); Linear curved vessels: unspecific distribution (4/9); white patchy scales (6/9); Structureless areas: focal orange (6/9); focal brown (4/9), focal white (1/9), diffuse orange (1/9); brown reticular (3/9), white dots (1/9), brown dots (1/9), white globules (1/9)
	Soliman et al. [12]	Cross-sectional study	Dermlite II PRO HR (3Gen) using polarized light, magnification ×10	88 patients; 32 patch stage classical, 16 with both patch and tumor stage classical, 20 hypopigmented, 8 poikilodermatous, 4 folliculotropic, 4 erythrodermic, 4 hyperpigmented	Non-homogeneous background (4/4), patchy whitish scales (4/4), pink to erythematous background (4/4), patchy areas of orange discoloration (4/4), patchy red globules (4/4), dotted vessels (4/4), short linear vessels (4/4), spermatozoa-like vessels (4/4), arborizing vessels (4/4)
	Ali et al. [1]	Prospective cross-sectional study	Dermlite DL4,3 Gen, Inc, San Juan Capist- rano, CA, USA with ×10 magnification	53 patients; 10 patch stage classical, 9 plaque stage classical, 3 tumor stage classical, 16 hypopigmented, 7 hyperpigmented, 5 poikilodermatous, 3 erythrodermic	Vascular changes: fine linear short vessels (3/3), dotted vessels (2/3), polymorphous pattern (2/3), purpuric spots (2/3); Scales: white (3/3), geometric white (3/3), perifollicular white (2/3), lamellar (1/3); Color changes: orange yellowish patches (2/3), white structureless patches (2/3), whitish pink structureless areas (2/3)
	Sławinska et al. [26]	Case–control study	FotoFinder videodermoscope (non-polarized dermoscopy; video camera Medicam 800HD) initially without immersion to evaluate the color and distribution of a scale/crust and subsequently with ultrasound gel as an immersion fluid (×20 magnification)	28 patients with erythroderma (9 MF, 8 atopic dermatitis, 3 SS, 3 pityriasis rubra pilaris, 1 allergic eczema, 1 dermatomyositis sine myositis, 1 psoriasis, 1 actinic reticuloid, 1 crusted scabies)	**Dermoscopic features** Vessels: dotted (9/9), linear (9/9), linear with branches (1/9), linear curved (1/9); Vessel distribution: unspecific (9/9); Scale color: white (9/9), Scale distribution: patchy (9/9); pink structureless areas (1/9); white-pinkish structureless areas (9/9); orange structureless areas (1/9); brown structureless areas (7/9) **Trichoscopic features** Vessels: linear (5/9), dotted (5/9); Vessel distribution: unspecific (5/9); Scale color and distribution: white interfollicular (6/9), yellow interfollicular (2/9); Follicular findings: perifollicular pigmentation (2/9), interfollicular pigmentation (3/9); red structureless areas (1/9); pink structureless areas (1/9); white-pinkish structureless areas (6/9); gray dots (1/9)
	Golinska et al. [27]	Case–control study	Trichoscopy (Medicam 1000; FotoFinder Systems GmbH, Bad Birnbach, Germany) was performed in two modes (with and without ultrasonography gel used as immersion fluid) at 20–70-fold magnification	49 patients with erythroderma (15 atopic dermatitis, 7 MF, 6 allergic contact eczema, 5 psoriasis, 4 SS, 4 drug reaction, 3 pityriasis rubra pilaris, 2 dermatomyositis, 1 actinic reticuloid, 1 crusted scabies, 1 pemphigus foliaceus)	Scale color: white (7/7), yellow (3/7); Scale distribution: interfollicular (7/7); Vessel arrangement: irregular (7/7); Vessel type: dotted (6/7), short linear irregular (5/7), spermatozoa-like (5/7), glomerular (1/7), branched (1/7), serpentine (1/7); whitish-pinkish structureless areas (7/7); orange structureless area (1/7); interfollicular pigmentation (4/7); perifollicular pigmentation (3/7)
	Rakowska et al. [28]	Case–Control Study	Trichoscopic scan with Fotofinder digital dermoscope (at a 20- and 70-fold magnification)	56 patients with erythroderma (16 MF or SS, 20 psoriasis, 20 atopic dermatitis), 20 healthy volunteers	Hair shaft: Numerous pili torti (13/16), visible anagen bulbs (2/16), 8-shaped hairs (3/16), solitary pili torti (2/16), numerous broken hairs (12/16), solitary broken hairs (3/16); Follicular ostia: yellow dots (14/16), black dots (4/16); Vessels: linear perifollicular (5/16), glomerular perifollicular (8/16), glomerular regularly distributed (8/16), dotted (4/16), arborizing (5/16), glomerular line arranged (2/16); Background: white thick interfollicular bands (9/16), patchy hyperpigmentation (6/16), reddish areas (12/16); Scale: yellow interfollicular (4/16), white interfollicular (14/16), follicular spicules-like (2/16)
	Batra et al. [49]	Observational cross-sectional study	Heine Delta II Dermatoscope with 10× magnification in polarized mode	29 patients with erythroderma (8 psoriasis, 5 endogenous eczema, 4 pityriasis rubra pilaris, 3 pustular psoriasis, 2 drug rash secondary to antitubercular therapy, 2 dermatophytic infection, 1 atopic dermatitis, 1 crusted scabies, 1 pemphigus foliaceus, 1 DRESS, 1 MF)	Background color: Dull erythematous with bluish-gray globules and structureless white areas in patchy distribution; Pattern of scaling: patchy; Color of scale: white; Character of scale: loose; Vessel character: combination of dotted and irregular vessels
	Errichetti et al. [50]	Case series	DermLite DL3; 3Gen, San Juan Capistrano, CA, USA	4 patients with erythroderma (1 psoriasis, 1 atopic dermatitis, 1 MF, 1 pityriasis rubra pilaris	Sparse white scales, widespread dotted vessels, and serpiginous (spermatozoa-like) vessels, with a whitish-pinkish background
**Pigmented Purpuric Dermatosis Like Mycosis Fungoides**	Aryal et al. [51]	Case report	NR	1 patient with pigmented purpuric dermatosis-like MF	Fine, short linear vessels; dotted vessels; orange-yellow patchy areas
	Nasimi et al. [52]	Case–control study	Dermoscopic photographs with FotoFinder Medicam 1000 (FotoFinder Systems GmbH, Bad Birnbach, Germany)	28 cases with PMF and 13 cases with PPD	PMF: Distributed dotted vessels (18/28) and spermatozoa-like structures (14/28), distributed in a cluster distribution (14/28) over a background of orange-yellowish (20/28) patchy areas, roughly with no scales (19/28) Spermatozoa-like structures (*p* = 0.014) and fine short linear vessels (*p* = 0.017) were significantly more frequent in PMF.
**Pagetoid Reticulosis**	Manoli et al. [53]	Case report	NR	74-year-old male patient	Regularly distributed dotted and glomerular vessels, pinkish background, coalescent whitish halos with a central red dot, diffuse hyperkeratosis with white scales
	Morariu et al. [54]	Case report	NR	50-year-old female patient	Homogenous pink background with erythema and dotted glomerular vessels, and white areas corresponding to scales, a negative network at the periphery
	di Meo et al. [55]	Case report	NR	66-year-old male patient	Structureless pinkish area in the central, segmental brown radial lines, large scaly circular peripheral border with some linear vessels
	Tian et al. [56]	Case report	NR	44-year-old male patient	A homogeneous pinkish area with locally distributed structureless, whitish network and glomerular, rod-shaped, and some linear blood vessels

**Abbreviations:** MF, mycosis fungoides; NR, not reported; FMF, folliculotropic mycosis fungoides; SS, Sezary syndrome; LPP, lichen planopilaris; DRESS, drug reaction with eosinophilia and systemic symptoms; PMF, purpuric mycosis fungoides; PPD, pigmented purpuric dermatosis.

### 3.3. Reflectance Confocal Microscopy in Mycosis Fungoides

**Figure 2:**
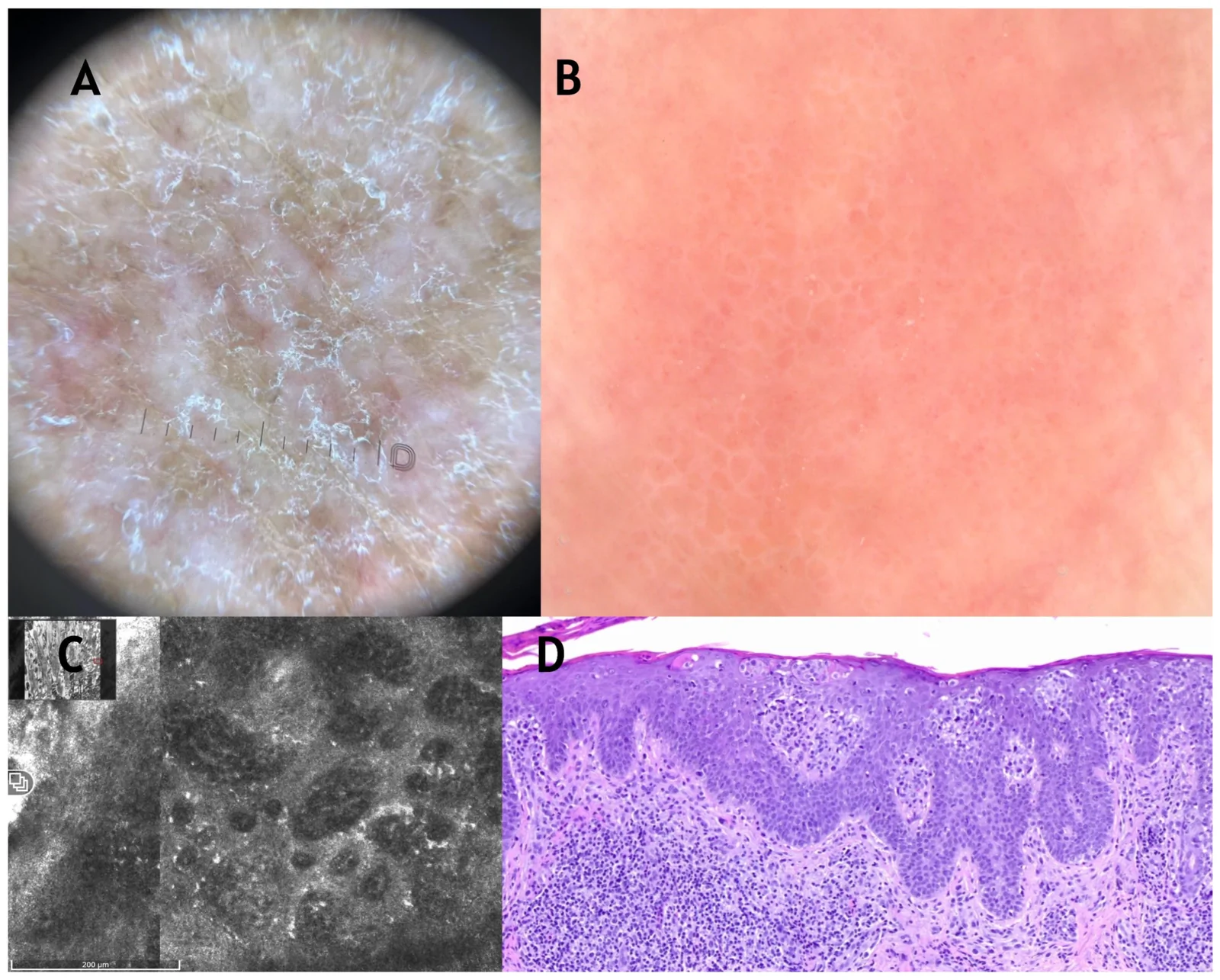
Representative dermoscopic, reflectance confocal microscopy (RCM), and histopathological findings in mycosis fungoides. (**A**) Dermoscopic image of a plaque-stage MF lesion showing orange-yellow structureless areas, patchy white scaling with scales accentuated in the skin furrows, and a pink-to-erythematous background. (**B**) Dermoscopic image of a tumor-stage MF lesion showing red globules separated by white lines and scattered spermatozoa-like vessels. (**C**) RCM image of the tumor-stage lesion showing architectural disarray, hyporefractile dermal papillae, scattered round-to-oval weakly refractile cells compatible with junctional/epidermotropic lymphocytes and Pautrier microabscess. (**D**) Corresponding histopathological image showing epidermotropism and intraepidermal collections of atypical lymphocytes consistent with Pautrier microabscesses.

RCM enables horizontal, in vivo visualization of the epidermis and superficial dermis at cellular resolution. Although the images are rendered in grayscale at the histological level, structures with high refractive indices, such as melanin and keratin, appear bright, whereas the cell nucleus—which has a low refractive index—appears dark [3]. Representative dermoscopic, RCM, and corresponding histopathological images of MF are presented in Figure 2.

In our review, we identified 17 studies evaluating MF skin lesions using RCM. All studies are presented in Table 4. The first study evaluating RCM in MF was conducted by Agero et al. and included 7 MF patients (3 patch, 4 plaque, and 1 tumor lesion). In patch lesions, the study demonstrated epidermal architectural disarray characterized by thickening and blurring of intercellular connections at the epidermal level. These areas corresponded histopathologically to mild spongiosis. Concurrently, scattered oval-to-round, weakly refractile cells, corresponding histopathologically to epidermotropic lymphocytes, were detected in the spinous layer. At the dermoepidermal junction, the basal cells surrounding the dermal papillae appeared as hyporefractile rings, in contrast to the bright cells observed in confocal examination of normal individuals. Evaluation at the dermal level was not feasible due to reduced contrast and loss of detail. In plaque lesions, the confocal findings were more pronounced. Dark, vesicle-like spaces filled with oval-to-round, weakly refractile cells were detected, extending from the superficial epidermal layers to the basal layer. These structures corresponded histopathologically to Pautrier microabscesses. Additionally, prominent and dilated blood vessels were observed. Other observed findings were similar to those in patch lesions but more pronounced. The tumor lesion exhibited epidermal disarray, oval-to-round, weakly refractile cells in the epidermis (some of which were large), and hyporefractile rings in the dermal papillae, consistent with other MF stages. However, the deep nodular atypical lymphocyte clusters observed histopathologically could not be visualized due to the limited penetration depth of RCM [57]. Li et al. conducted a study of 10 MF patients examining the relationship between confocal microscopy features and histopathological findings, and their results were consistent with prior research [58].

Following the establishment of RCM criteria for MF, a study assessed the distribution, sensitivity, and specificity of these findings, along with inter-observer agreement in their identification. In this study, both lesional and normal skin areas were examined by a primary investigator and two blinded secondary assessors. Findings demonstrating significant inter-observer reproducibility included junctional atypical lymphocytes, architectural disarray, spongiosis, hyporefractile papillae, and blood vessel dilatation. Epidermal atypical lymphocytes, loss of demarcation, and dendritic cells, while not as prominent as the previous findings, were among the features that showed homogeneity among the researchers. However, inter-observer agreement was poor for vesicle-like structures, fibrosis, dermal atypical lymphocytes, and keratinocytes with elongated nuclei. The presence of spongiosis (94.7% sensitivity/88.9% specificity for investigator 1; 100% sensitivity/77.8% specificity for investigator 2), loss of demarcation (thickening or blurring of intercellular spaces, ill-defined cell borders) (94.7%/88.9% for investigator 1; 94.7%/77.8% for investigator 2), and disruption of epidermal architecture (89.5%/77.8% for investigator 1; 100%/77.8% for investigator 2) were identified as features with high sensitivity and specificity. Spongiosis, epidermal disarray, presence of hyporeflective papillae, and epidermal atypical lymphocytes were the most frequently observed features [59].

In a 2016 study by Mancebo et al., which evaluated 83 lesions from 43 MF patients, folliculotropic MF and hyperpigmented MF subtypes were assessed in addition to classic MF. Morphological differences were observed in patch lesions, particularly at the dermoepidermal junction and papillary dermis. The study reported that hyperpigmented patches more frequently exhibited hyperreflective dermal papillary rings, in contrast to the hyporeflective papillary rings observed in erythematous patches. Additionally, dermal melanophages and lymphocytic infiltration at the dermoepidermal junction were reported more frequently than in erythematous patches, while erythematous patches were characterized by increased blood vessel dilatation and fibrosis of the papillary dermis. Furthermore, in addition to findings from other studies, perivascular infiltration in the papillary dermis was identified in patch lesions. In plaque lesions, Pautrier microabscesses and epidermal dendritic processes were reported as characteristic features. Pautrier microabscesses were also observed in tumoral lesions. It was noted that the structure and arrangement of papillary rings were disrupted at the dermoepidermal junction of tumoral lesions. In folliculotropic MF, lymphocytes infiltrating and surrounding the follicular epithelium, and mucin deposition within the hair follicle, were observed. RCM examination revealed that the presence of Pautrier microabscesses was associated with higher rates of TCR monoclonality positivity and elevated MF histopathological scores [60].

Zhu and colleagues evaluated 60 patients with suspected early-stage MF and chronic inflammatory skin diseases using RCM and histopathology, and observed that 8 of 12 patients diagnosed with MF according to International Society for Cutaneous Lymphomas histopathology criteria also demonstrated typical MF features on RCM. They found statistically significant concordance between the histopathological and RCM findings [61].

Melhoranse Gouveia et al. evaluated 38 MF lesions at diagnosis and 6 months thereafter to assess the utility of confocal microscopy in the follow-up of MF. They determined that the strongest correlation between histopathology and confocal characteristics was observed for Pautrier microabscesses, epidermal and junctional lymphocytes, and interface dermatitis/hyporeflective papillae. Subsequently, they reported an area under the curve of 0.95 for evaluating disease severity using a combination of these findings. It was reported that the scoring of this combination demonstrated excellent correlation with clinical evaluation, increasing in progressive lesions and decreasing in lesions showing clinical improvement [62]. In another study by the same group, 6 lesions from 3 patients with suspected large cell transformation were evaluated using RCM. In 5 lesions where histopathology confirmed large cell transformation, numerous large, bright, round, pleomorphic cells, approximately 20 μm in diameter, potentially corresponding to large lymphocytes, were detected at the dermoepidermal junction and upper dermis. These distinctive cells were not observed in the single lesion without a histopathologically confirmed large cell transformation [63].

In a study assessing the efficacy of RCM in diagnosing and differentiating erythematosquamous skin diseases, three independent dermatologists with moderate confocal microscopy experience examined 1700 RCM images comprising cases of MF, psoriasis, contact dermatitis, chronic and subacute lupus, and healthy controls. They were instructed to determine diagnoses and identify the presence or absence of previously established confocal features. The evaluation demonstrated a sensitivity of 63.33% and specificity of 92.89% for MF. The study also identified epidermotropic atypical lymphocytes, interface dermatitis, pleomorphic tumor cells, and dendritic cells as specific confocal features of MF lesions. The study indicated that the principal factor contributing to the observed low sensitivity in MF was that epidermotropic atypical lymphocytes and interface dermatitis may also be observed in cutaneous lupus, potentially resulting in a mix-up between the two conditions [64]. In another study evaluating the role of confocal microscopy in the differential diagnosis of hypopigmented lesions, lesions showing complete absence of melanin in the basal layer on RCM images were histopathologically consistent with vitiligo. In contrast, lesions showing partial melanin loss in the basal layer—characterized by monomorphic, weakly refractile, oval-to-round cells within vesicle-like dark spaces in the epidermis, along with weakly refractile round-to-oval cells scattered within the papillary dermis or dermal papillary rings—were histopathologically consistent with MF. The study demonstrated excellent concordance between RCM and histopathological findings [65].

**Table 4:** Studies involving the evaluation of MF skin lesions using reflectance confocal microscopy.

Studies	Study Objective	Patient Group	Results
Agero et al. [57]	To evaluate MF lesions using RCM and to correlate RCM features with histopathology	8 MF lesions from 7 patients (3 patch, 4 plaque, 1 tumor)	In the spinous layer, oval to round weakly refractile cells, epidermal architectural irregularities, thickened and blurred intercellular demarcations, and epidermal cells with elongated nuclei At the dermoepidermal junction, basal cells appeared as faintly refractile rings surrounding the dermal papillae Plaque-type lesions: In addition to others, vesicle-like dark spaces filled with monomorphic oval to round cell clusters with poor refractive index
Koller et al. [64]	To evaluate the diagnostic accuracy of confocal imaging of erythematosquamous skin diseases, to identify typical RCM features, and assess their diagnostic performance	1700 RCM images from 75 patients with psoriasis (27 patients), contact dermatitis (20 patients), MF (10 patients, including two patch-type, six plaque-type, and two tumor-type), CDLE (4 patients), or SCLE (14 patients), and from 10 healthy adults	The sensitivity and specificity values for MF were found to be 63.33% and 92.89%, respectively; for psoriasis, 89.13% and 95.41%; for contact dermatitis, 83.33% and 92.31%; and for SCLE/CDLE, 62.96% and 94.53%. MF features in RCM: Epidermotropic atypical lymphocytes, interface dermatitis, Pleomorphic tumor cells and dendritic cells were found to be specific
Lange-Asschenfeldt et al. [59]	To test the applicability of RCM in the diagnosis and differential diagnosis of MF, and to evaluate the concordance of findings among confocal practitioners	39 test sites from 15 patients: 10 MF, 3 parapsoriasis, 1 SS, 3 Lyp (2 patients had both MF and Lyp), 9 sites were normal skin sites from the same patients	The most frequent RCM features: Spongiosis (94.7%), loss of demarcation (94.7%), epidermal disarray (89.5%). The highest inter-observer reproducibility: Junctional atypical lymphocytes, architectural disarray, and spongiosis Features exhibiting high sensitivity/specificity: Spongiosis, loss of demarcation, and disruption of epidermal architecture
Ardigo et al. [2]	To demonstrate the usefulness of confocal microscopy in evaluating therapeutic response in patients with cutaneous T-cell lymphoma affecting the scalp and causing scarring alopecia.	1 patient with FMF	Parakeratosis in the stratum corneum, Inflammatory cells as bright structures in the stratum spinosum, single to aggregates of bright cellular structures between thickened dermal fibers as lymphocytic infiltrates in the upper dermis and thickening of the dermal fibers in the dermis After treatment: Mild infiltrate of cells located around the adnexal structures’ ostium in the spinous layer, lymphoid cells inside the adnexal epithelium in the upper dermis. In the dermis, around the adnexal structures, massive infiltration of inflammatory cells
Li et al. [58]	To define the RCM characteristics of MF and evaluate its applicability in biopsy site selection.	10 MF patients (3 patch stage, 5 plaque stage, and 2 tumor stage)	The identified RCM features were found to correlate well with histopathological evaluation. Hyperkeratosis: Five patients (50%) Disarray in the honeycomb structure of the stratum spinosum: 3 patients (30%). Weak light-reflecting dermal papillary rings: All patients Widespread round to oval cells in the epidermis and papillary dermis, and inflammatory cell infiltration in the superficial dermis: All patients Vesicle area opaca (filled with monomorphous weakly refractile oval to round cells): 2 patients (20%)
Mancebo et al. [60]	To evaluate the morphological features of MF/SS using RCM and the correlation of RCM features with histopathology and TCR gene rearrangement studies	A total of 83 lesions: 56 patches, 20 plaques, 3 papules, 1 tumor, and 3 erythrodermic skin lesions (72 lesions corresponded to 36 patients with MF, 8 lesions to 3 patients with folliculotropic MF, and 3 lesions to 2 patients with SS)	- The most frequently observed RCM features were epidermal lymphocytes (86%), epidermal disarray/loss of demarcation of keratinocytes (88%), and blood vessel dilatation (79%). - Erythematous patch: Epidermal lymphocytes (83%), epidermal disarray (83%), hyporeflective dermal rings (97%), blood vessel dilatation (88%), Pautrier collections (41%), perivascular infiltrate (16%) Hyperpigmented patch: Epidermal lymphocytes (79%), epidermal disarray (84%). Hyper-reflective dermal papillae rings (73%), lymphocytes at DEJ (68%), mixed infiltrate (63%) Plaque lesions: Pautrier collections (80%), epidermal lymphocytes (95%), epidermal disarray (95%), dendritic processes (60%), hyporeflective dermal rings (90%), blood vessel dilation (85%) Folliculotropic MF: Lymphocytes surrounding and penetrating the follicular epithelium (100%) and mucin deposition (50%) in the hair follicle. The highest correlation between RCM and histopathology: Epidermal lymphocytes and melanophages in the dermis.
Yeager et al. [66]	Presenting the RCM findings of tumoral MF	1 patient (Tumor stage)	Disarray of the epidermis with small, weakly refractile, round to oval cells scattered within the spinous layer and dermoepidermal junction. Vesicle-like dark spaces filled with weakly refractile, round to oval cells and hyporefractile basal cells surrounding the dermal papillae.
Fabbrocini et al. [67]	To report the current utilization of RCM as a guide for skin biopsy	1 patient (FMF)	Small, weakly refractile round to oval cells in the epidermis, a disorganized honeycomb pattern, thickening of dermal fibers, and inflammatory cells in the upper dermis. Round to oval refractile cells around and within the hair follicle
Porto et al. [68]	To present the role of RCM in the diagnosis of MF	1 MF patient	Epidermal disarray with the infiltration of bright cells and hyporeflective papillae at the DEJ
Zhu et al. [61]	To analyze the consistency of pathological and RCM features in early-stage MF and the usefulness of RCM in the diagnosis of early-stage MF.	60 patients (40 suspected MF cases and 20 clinically diagnosed chronic inflammatory skin diseases)	There was a strong consistency between the classification by RCM and the diagnosis algorithm. RCM findings showing significant consistency with histopathology: Patch stage: Epidermal lymphocytes, epidermal disarray, perivascular lymphocyte infiltration, fibrosis Plaque stage: Pautrier microabscess, dermal lymphocytes, fibrosis The main RCM findings of the patch stage: Epidermal disarray, weakly refractile, round to oval atypical lymphocytes in the epidermis and DEJ. Perivascular lymphocyte infiltration and fibrosis in dermis. The main RCM findings of plaque stage: The destruction of the honeycomb structure of the stratum spinosum, atypical lymphocytes in the epidermis to dermis, and Pautrier microabscesses. Perivascular lymphocyte infiltration and fibrosis in the dermis
Melhoranse Gouveia et al. [62]	To evaluate the level of agreement between RCM and histopathology and to develop a RCM checklist that can aid in the follow-up of MF patients.	38 MF lesions	- RCM findings showing high agreement with histopathology: Weakly bright, round to oval epidermal lymphocytes (78% OPA), Pautrier microabscesses (vesicle-like structures with weak reflective round cells inside) (90% OPA), junctional lymphocytes (weakly refractile, round to oval cells at the DEJ) (71% OPA), hyporefractile papillae (66% OPA), dermal lymphocytes (64% OPA). Epidermal irregularity was not scored by the pathologist and therefore not included in the analysis, as it is specific to the horizontal assessment of RCM. - The combination of the four RCM parameters with the highest OPA values (epidermal and junctional lymphocytes, Pautrier microabscesses, and interface dermatitis/hyporefractible papilla) has an AUC value of 0.95 in predicting disease severity
Melhoranse Gouveia et al. [63]	To assess lesions suggestive of large cell transformation using RCM	3 patients (6 lesions)	In five lesions where large cell transformation was detected in pathology, numerous large, bright, round, pleomorphic cells, approximately 20 μm in diameter, were identified at the dermoepidermal junction/upper dermis.
Farabi et al. [69]	To describe the typical presentation of a plaque-stage MF case in RCM and to examine the relevant histological findings.	1 patient (plaque stage MF)	In the upper layers of the epidermis: Typical honeycomb structure and parakeratosis At the level of the stratum spinosum: Vesicle-like dark spaces filled with monomorphic, weakly refractile, large oval to round nucleated cell clusters (as the depth of the epidermis increased, the diameter of the vesicle-like spaces increased, and monomorphic hypo-refractile cell clusters were observed along with larger atypical hyper-refractile cells) At the DEJ level: Large, atypical cells with dendritic morphology, minimal inflammation, and well-defined hypo-refractile papillae In the deeper layers of the dermis: The same vesicular structures with hypo-refractile borders that decrease in size with increasing depth
Liu et al. [65]	To investigate the effectiveness of RCM in the differential diagnosis of HMF and vitiligo	15 patients (13 patients with HMF, 2 with vitiligo)	Vitiligo: Absence of melanin HMF: Slight reduction in melanin in the basal layer, monomorphic, weakly refractile, oval-round cells within vesicle-like dark spaces in the epidermis and weakly refractile, round-oval cells scattered within the papillary dermis and dermal papillary rings
Tian et al. [56]	To report dermoscopic and RCM clues of Pagetoid Reticulosis	1 patient with pagetoid reticulosis	Small and weakly refractile round or oval cell infiltrations in the spinous layer, dermal papilla, and superficial dermis, non-edged (hyporefractile) dermal papillae, and increased dendritic cells in the basal layer
Zha et al. [48]	To present a patient who previously followed up with lichen planus pigmentosus and subsequently developed hyperpigmented MF	1 patient with hyperpigmented MF	Thickening of the spinous layer, liquefaction degeneration of basal cells, vasodilation in the superficial dermis, and infiltration of bright inflammatory cells
D’Onghia et al. [70]	To describe dermoscopic, RCM, and LC-OCT features of PCL (CTCL and CBCL) and to explore their role in PCL management	A total of 40 lesions, including 30 CTCL (19 MF, 8 SS, 2 primary cutaneous aggressive epidermotropic CD8+ cytotoxic T-cell lymphoma, and 1 PCALCL) and 10 CBCL, of 25 patients	Atypical epidermal architecture: LC-OCT: 50%, RCM: 66.6% Epidermotropism: LC-OCT: 73.1%, RCM:72.2% Pautrier’s microabscess: LC-OCT: 3.8% RCM: 22.3% Spongiosis: LC-OCT: 7.7% RCM: 22.2% Interface dermatitis/non-edged papillae: LC-OCT: 28% RCM: %16.7 Junctional lymphocytes: LC-OCT: 66.7% RCM: 55.6%

**Abbreviations:** MF, mycosis fungoides; RCM, reflectance confocal microscopy; CDLE, chronic discoid lupus erythematosus; SCLE, subacute cutaneous lupus erythematosus; SS, Sezary syndrome; TCR, T-cell receptor; DEJ, dermoepidermal junction; OPA, overall per cent of agreement; AUC, area under the curve; HMF, hypopigmented mycosis fungoides; PCL, primary cutaneous lymphoma; CTCL, cutaneous T-cell lymphoma; CBCL, cutaneous B-cell lymphoma; PCALCL, primary cutaneous anaplastic large cell lymphoma; LC-OCT, line-field confocal optical coherence tomography.

### 3.4. High-Frequency Ultrasound in Mycosis Fungoides

HFUS designates ultrasonic imaging modalities operating at frequencies of 20 MHz and above, which facilitate visualization of cutaneous structures and skin appendages. As frequency parameters increase, spatial resolution and morphological detail are enhanced; however, the depth of tissue penetration becomes correspondingly more superficial [71].

In our review, we identified nine studies that included the evaluation of MF skin lesions using HFUS. These studies are presented in Table 5. In 2017, Polanska et al. performed the first assessment of UVA1 and PUVA therapeutic effects on 18 MF patients using HFUS. They documented that the subepidermal low echogenic band (SLEB) demonstrated progressive attenuation following treatment, with complete resolution observed in some patients. Furthermore, they established a positive correlation between SLEB thickness and disease severity; SLEB thickness increased proportionally with advancing disease stage [72]. The same authors subsequently reported a 5-year follow-up evaluation of 3 early-stage MF patients (19 lesions) utilizing ultrasound. They documented complete resolution of SLEB in patients achieving complete remission, whereas those demonstrating partial response exhibited attenuation of SLEB without complete disappearance [73].

A study involving 10 MF patients assessed T cell infiltration histopathologically using HFUS. In this study, the mean SLEB thickness (parameter 1) and the sum of the mean SLEB and the thickness of the entry echo (parameter 2) were measured. Concurrently, the mean subepidermal thickness of T cells (microscopic parameter 1) and the thickness of subepidermal infiltration of T cells together with the epidermis (microscopic parameter 2) were measured histopathologically. A correlation was identified between parameter 1 and microscopic parameter 1, as well as between parameter 2 and microscopic parameter 2. Furthermore, the study found that SLEB is thicker in plaque lesions compared to patch lesions [74]. A comparative study examined the HFUS features of early-stage (patch and plaque) and advanced-stage (tumor) MF. The study demonstrated that early-stage lesions exhibited more homogeneous internal echogenicity, whereas advanced-stage lesions were characterized by increased infiltration depth, unclear SLEB boundaries, and the presence of echogenic foci; these findings were deemed statistically significant. Additionally, two FMF lesions were evaluated in the same study, revealing patchy hypoechoic areas with unclear boundaries surrounding the hair follicles, extending toward the epidermis, and associated with SLEB [75]. A patient with MF resistant to topical corticosteroids and phototherapy was initiated on chlormethine gel treatment, and the lesion was evaluated before and after treatment using ultra-high-frequency ultrasound (UHFUS) (70 MHz). Before treatment, the thick hyperechogenic layer corresponding to the stratum corneum measured 0.18 mm, and the SLEB measured 0.94 mm. In C-mode, numerous dilated hair follicles with increased vascular signal were observed, disrupting the dermo-epidermal junction. Numerous vascular lacunae were also noted at the dermal level. After treatment, the epidermis measured 0.11 mm, and the SLEB measured 0.15 mm, with a significant decrease in the thickness of both layers and in the vascular signal observed [76]. One study evaluated whether the change in SLEB varied according to gender, age, and type of treatment. In this study, which included 31 MF patients, the reduction in SLEB did not differ according to systemic or topical treatment or according to gender. While a significant reduction was observed in older patients, the reduction with treatment was not statistically significant in younger patients [77].

**Table 5:** Studies involving the evaluation of MF skin lesions using high-frequency ultrasound.

Studies	Study Objective	Patient Group	Frequency of the US	Results
Polanska et al. [72]	To evaluate the effectiveness and clinical response of UVA1 and PUVA therapy in the treatment of MF patients using HFUS	18 MF patients (stages I–IIA), 6 of them underwent UVA1 phototherapy, 12 of them underwent PUVA phototherapy	20 MHz	All subjects demonstrated SLEB in the lesional skin. SLEB thickness decreased with phototherapy. In patients with more advanced disease, the SLEB was thicker.
Polanska et al. [73]	To present the benefit of 20 MHz HFUS in objectively monitoring treatment response for 5 years in MF patients.	3 early-stage MF patients (19 lesions) were followed up for 5 years. 2 patients received PUVA, UVA1, and topical corticosteroid therapies, while 1 patient received PUVA and topical corticosteroid therapies.	20 MHz	In cases of partial response, SLEB demonstrated thinning while residual SLEB remained detectable. In cases of complete response, SLEB was entirely absent.
Polanska et al. [74]	To demonstrate the characteristic high-frequency ultrasonographic features of MF in relation to histopathological findings.	10 early-stage MF patients	20 MHz	SLEB demonstrated correlation with subepidermal infiltration of clonal T cells
Wang et al. [75]	To evaluate the ultrasound characteristics of MF/SS and to investigate the value of HFUS in the accurate staging of cMF	26 MF patients: 23 classic MF, 2 FMF, 1 SS Among the classic MF cases, 5 were in the patch stage, 11 in the plaque stage, and 7 in the tumor stage	50 and 20 MHz	Early-stage lesions displayed homogeneous internal echoes, whereas advanced-stage lesions were characterized by increased infiltration depth, unclear SLEB boundaries, and the presence of echogenic foci. In FMF lesions, patchy hypoechoic areas around the hair follicles were detected
Niu et al. [78]	To evaluate the value of HFUS in the differential diagnosis of early-stage MF and inflammatory skin diseases	62 patients (18 early-stage MF and 44 age-matched patients with psoriasis vulgaris and eczema)	50 MHz	SLEB and epidermal thickness were found to be influential factors in differential diagnosis. In the early-stage MF group, epidermal thickness and SLEB thickness were significantly less compared to those of inflammatory diseases. Sensitivity and specificity for early-stage MF were obtained as 55.6% and 93.2%, respectively, when epidermal thickness was <0.2375 mm and SLEB thickness was <0.2655 mm.
Wieser et al. [20]	To prospectively evaluate skin lesions in patients with CTCL lesions using dermoscopy and HFUS, and to compare the findings with AD and psoriasis	13 patients: 6 CTCL, 5 psoriasis (1 patient was only evaluated with dermoscopy due to technical issues), and 2 atopic dermatitis CTCL: 3 patients had only patch lesions and were in stage IB (2 with classic MF, 1 with folliculotropic MF), 1 patient was in stage IB with both patch and plaque lesions, and 2 patients had Sézary Syndrome.	70 MHz transducer with a central wavelength of 50 MHz and dermoscopy (DL4)	Patients with CTCL and psoriasis had an increased epidermal thickness compared to AD; however, this was not significant due to the limited sample size. Psoriasis patients had a slightly increased dermal thickness compared with the CTCL patients (not significant). An uneven SLEB was detected in all patients.
Janowska et al. [76]	Evaluating the response to chlormethine treatment in an MF patient using HFUS	1 patient (Stage IB MF with patches and plaques) was treated with chlormethine gel	70 MHz	Post-treatment, a decrease in epidermal and SLEB thickness and vascular signaling were observed.
Cison et al. [79]	Sharing HFUS findings of a patient diagnosed with erythrodermic MF	1 patient (Erythrodermic MF)	22.5 MHz	HFUS was performed on the skin lesion and the normal skin. Skin lesion revealed a 0.1 mm hypoechoic band below the entry zone.
Cison et al. [77]	To evaluate the usefulness of HFUS in detecting changes in SLEB thickness in relation to treatment	31 MF patients (22 patients received systemic treatment, 9 received topical treatment)	22.5 MHz	SLEB showed a statistically significant reduction after treatment. The reduction in SLEB did not differ according to systemic or topical treatment, or by gender. While a significant reduction was observed in older age groups, the reduction with treatment was not statistically significant in younger age groups.

**Abbreviations:** UVA1, ultraviolet A-1; PUVA, psoralen+UVA; MF, mycosis fungoides; HFUS, high frequency ultrasound; SLEB, subepidermal low-echogenic band; cMF, classic mycosis fungoides; FMF, folliculotropic mycosis fungoides; SS, sezary syndrome; AD, atopic dermatitis; CTCL, cutaneous T-cell lymphoma.

A study evaluating the differentiation of early-stage MF lesions from inflammatory skin diseases using HFUS examined 18 patients with early-stage MF and 44 age-matched patients with psoriasis and eczema. The study demonstrated that SLEB and epidermal thickness were significantly lower in early-stage MF compared to inflammatory diseases. For early-stage MF, the epidermal thickness measured <0.2375 mm and the SLEB thickness measured <0.2655 mm, with sensitivity and specificity of 55.6% and 93.2%, respectively. The study also revealed that the number of epidermal linear acoustic shadows was significantly greater in psoriasis lesions than in early-stage MF and eczematous skin lesions [78]. In another study involving 13 patients, 6 of whom had CTCL (MF + SS), the mean epidermal, SLEB, and dermal thicknesses of MF, psoriasis, and atopic dermatitis lesions were compared. In contrast to the previous study, epidermal thickness was found to be greater in CTCL (MF + SS) and psoriatic lesions compared to atopic dermatitis, although this difference did not reach statistical significance due to the limited sample size. Dermal thickness was also found to be greater in psoriatic patients compared to CTCL patients [20].

### 3.5. Line-Field Confocal Optical Coherence Tomography in Mycosis Fungoides

LC-OCT allows real-time in vivo imaging in horizontal, vertical, and 3D formats and provides cellular-level resolution [80]. We identified two studies that evaluated MF skin lesions using LC-OCT. In 2023, Soglia et al. examined classic and folliculotropic MF lesions using LC-OCT. The study included 6 patches, 2 plaques, 1 tumor, and 1 folliculotropic MF lesion. In the early stages of MF, epidermal architectural irregularities were observed, attributable to focal disruption of the honeycomb pattern and spongiosis. Scattered atypical lymphocytes—round-to-oval, small, and weakly refractile—were also visualized within the epidermis, dermo-epidermal junction, and papillary dermis, aided by the technique’s substantial penetration depth. In plaque-type MF, these findings were further accompanied by more pronounced acanthosis and Pautrier microabscesses, manifesting as aggregations of atypical lymphocytes organized into vesicle-like, round, dark cavities within the epidermis. For tumor-type MF, a dermal nodular component consisting of hyporefractive cells mixed with scattered hyperrefractive cells was identified within the dermis. The superiorly displaced epidermis demonstrated indistinct demarcation from the underlying nodular component. In folliculotropic MF, hyperrefractive, oval-to-elongated cells were mainly seen within the follicular and perifollicular zones. Although hair shafts were occasionally not clearly discernible, the follicular ostia frequently exhibited a cystic appearance [81].

D’Onghia et al. conducted a comparative study of RCM and LC-OCT encompassing 30 cutaneous T-cell lymphoma lesions (19 MF, 8 SS) and 10 cutaneous B-cell lymphoma lesions. Hyperkeratosis and parakeratosis in the epidermis were detected at a statistically significantly higher frequency with LC-OCT than with RCM. Conversely, Pautrier microabscesses, spongiosis, and dendritic cells were identified more frequently with RCM, though this difference did not reach statistical significance [70].

### 3.6. Other Non-Invasive Imaging Techniques

Ring et al. examined lymphomatoid papulosis and MF lesions using OCT. The study included 2 lymphomatoid papulosis and 3 MF patients (1 plaque-stage MF and 2 tumor-stage MF). The study reported that for plaque-stage MF, the normal architecture of the skin was disrupted, the dermo-epidermal junction was indistinguishable, and the dermis had a hyperreflective homogeneous appearance. In one of the tumor-stage MF patients, a homogeneous, wavy-shaped formation of the superficial skin with fringes demarcated by a hyperreflective border was reported, while in the other, a sharply demarcated hyperreflective formation extending from the epidermis to the deeper part of the dermis was observed [82]. In one case report, a patch-stage MF lesion was evaluated with OCT, and a hyperreflective and thickened stratum corneum was reported. The papillary dermis showed a hyporeflective longitudinal structure, along with several more obscure dark structures deeper in the dermis [83].

In a further study [84], the combination of hyperspectral imaging and artificial intelligence (AI) was assessed for the differential diagnosis of MF from atopic dermatitis and psoriasis. The study comprised 1659 images, including 333 MF, 421 psoriasis (PsO), 351 atopic dermatitis (AD), and 554 normal skin images acquired using a digital camera and spectrometer. Employing a k-fold cross-validation value of 7, the model demonstrated a sensitivity of 90.72%, a specificity of 96.76%, an F1 score of 90.08%, and an ROC-AUC of 0.9351.

A study comprising 56 early-stage MF patients and 10 healthy controls evaluated the efficacy of multispectral imaging (MSI) and laser speckle contrast imaging (LSCI) in the diagnosis and treatment monitoring of the disease. In the study, MSI evaluated erythema (quantified by CIELAB a* and mean hemoglobin), pigmentation, plaque elevation, scaling, and individual typology angle (ITA), whereas LSCI measured microvascular perfusion and colorimetry quantified erythema (CIELAB a*). While all parameters successfully differentiated between healthy control skin, non-lesional skin, and lesional skin, those evaluating pigmentation, desquamation, and elevation did not reach statistical significance upon confirmation. Notably, MSI CIELAB a*, MSI mean hemoglobin, colorimetric CIELAB a*, and MSI ITA reliably distinguished lesional from non-lesional skin, demonstrated strong test–retest reliability, detected treatment-related changes, and showed moderate agreement with the Composite Assessment of Index Lesion Severity (CAILS). Furthermore, these markers were associated with a low patient burden, with a mean score of 3.4 out of 100 [85].

## 4. Discussion

MF is frequently diagnosed with considerable delay, particularly during its early stages, as it may closely mimic other erythematous conditions both clinically and histopathologically. A multicenter study documented a mean diagnostic delay of 36 months, although this interval may extend substantially longer in certain cases [86,87]. In addition to shortcomings in the diagnosis of MF, there remain areas requiring clarification in the staging of clinical lesions. The currently employed TNMB staging system defines a patch as any size skin lesion without significant elevation or induration; a plaque as any size skin lesion that is elevated or indurated; and a tumor as a solid or nodular lesion at least 1 cm in diameter demonstrating signs of depth and/or vertical growth. However, the same guideline acknowledges that distinguishing patch from thin plaque, and thick plaque from tumor, is highly subjective in practice. These distinctions are more than semantic: prognosis, treatment selection, and treatment response are all linked to how a lesion is classified [88]. One study assessed dermatologists’ agreement on the morphology of MF lesions, identifying 24 lesions as either patches or plaques. The study reported consensus on 67% of these lesions, whereas 33% remained discordant [89]. Additionally, three studies on folliculotropic MF reported that patch/thin plaque lesions demonstrated a more favorable prognosis, whereas thick plaque lesions exhibited more aggressive behavior, suggesting the need for a different staging system for FMF [90,91,92]. Following these studies, the terms “thin” and “thick” plaques gained further importance. In 2022, a survey was conducted among 22 specialists (pathologists, dermatologists, hematologists, and oncologists) who were members of EORTC and the International Society for Cutaneous Lymphomas, evaluating their opinions on plaque lesions. Only 33.3% of the questions yielded a full/very high degree of consensus regarding patch or thick plaques. In the clinical differentiation between thin and thick plaques, only 27.3% of participants believed this could be achieved through clinical examination alone. In conclusion, the study failed to reach a consensus on the precise definition of thin and thick plaques, and the current positioning of plaques within the TNMB system was considered clinically inadequate. It was noted that there is a substantial need for prospective studies evaluating the role of promising diagnostic tools, such as imaging modalities, to fully integrate current clinical definitions with more objective parameters [93]. Non-invasive imaging techniques represent a promising approach to addressing current limitations in the early diagnosis and accurate staging of MF. A comparative overview of the non-invasive skin imaging modalities evaluated in MF, including their principal characteristics, advantages, limitations, and clinical applications, is provided in Table 6.

Dermoscopy is a practical, cost-effective, and non-invasive imaging modality widely employed in dermatological practice. Studies included in this review consistently support a reproducible dermoscopic triad—linear vessels, orange-yellow structureless areas, and a dull erythematous background—as a characteristic pattern of early-stage mycosis fungoides. Additional frequently described findings include spermatozoa-like vessels and patchy dot vessels [5,6,15]. Of particular note is the distribution of scaling; the recurrent observation of scaling localized to skin furrows suggests a patterned epidermal involvement that may assist in differentiating MF from other inflammatory dermatoses [1,11]. Nevertheless, the reported frequency of individual findings varies considerably across studies. As disease stage advances and lesion depth increases, corresponding changes in dermoscopic features are observed. In plaque- and tumor-stage lesions, white structureless areas may become apparent, reflecting underlying fibrosis. Tumor-stage lesions have additionally been associated with linear branching vessels, ulceration, and red globules delineated by white lines [11]. Collectively, these findings indicate that dermoscopy can yield clinically meaningful information not only for diagnostic purposes but also for lesion characterization.

Dermoscopy has also demonstrated utility in identifying MF variants. A substantial body of literature, comprising both cohort studies and case reports, has focused on folliculotropic MF in particular. Consistently reported dermoscopic features of folliculotropic MF include follicular plugs, dilation of follicular openings, perifollicular accentuation, loss of terminal hairs, broken or dystrophic hairs, and reduced hair density [11,23,25]. In the study by Wang et al., yellow globules were identified dermoscopically in early-stage lesions that appear patch-like clinically yet demonstrate folliculotropism on histopathology, suggesting that dermoscopy may facilitate the early detection of folliculotropism- a finding with direct implications for disease management [24]. Despite the overall concordance across studies, variability in reported findings persists, attributable in part to assessments performed at differing anatomical sites and to the absence of standardized terminology. In erythrodermic MF, the dermoscopic features described in the literature appear less consistent. Variability has been observed in the frequency of orange-yellow amorphous areas, scaling patterns, and vascular morphology, which are frequently described in classical MF [26,27,49]; this likely reflects both biological heterogeneity and methodological differences between studies.

**Table 6:** Comparative overview of the non-invasive skin imaging modalities evaluated in MF.

Modality	Imaging Principle/Depth	Major MF-Associated Findings	Main Advantages and Clinical Applications	Main Limitations/Current Evidence
**Dermoscopy/trichoscopy**	Superficial optical imaging; no cellular-level or deep-tissue assessment	Linear/spermatozoa-like vessels, orange-yellow structureless areas, dull erythematous background, scales in furrows; follicular plugs, broken hairs, yellow dots, dilated follicular openings in FMF	Rapid, inexpensive and widely available; useful for **increasing clinical suspicion**, lesion/subtype characterization, and potentially biopsy-site selection	Findings overlap with inflammatory dermatoses and vary with phototype/subtype. **Relatively broad but heterogeneous evidence; not diagnostic without histopathology.**
**RCM**	En face cellular-level imaging; penetration ~200–250 µm	Epidermal disarray, epidermal/junctional lymphocytes, hyporefractile papillae, Pautrier collections and spongiosis	Near-histologic in vivo assessment; useful as an **adjunct to diagnosis**, for **biopsy-site selection**, and for disease-activity/treatment monitoring	Limited depth; overlap with inflammatory/interface dermatoses; imperfect sensitivity; limited assessment of lymphocyte atypia due to insufficient nuclear/intracellular detail. **Growing but heterogeneous evidence.**
**HFUS/UHFUS**	Cross-sectional acoustic imaging; depth and resolution are frequency-dependent	**SLEB**, which may correlate with superficial lymphoid infiltration and decrease with treatment; deeper/irregular infiltration in more advanced lesions	Rapid, quantitative and repeatable; best supported for **treatment monitoring**. May provide information on cutaneous infiltration depth and lesion-stage characterization	SLEB is non-specific. Diagnostic utility is inconsistent; evidence for depth/stage characterization is limited. **Evidence is strongest for treatment monitoring.**
**LC-OCT**	Cellular-level vertical, horizontal and 3D imaging; greater penetration than RCM	Epidermal disarray, atypical lymphocytes, Pautrier collections, and dermal/follicular changes	Combines cellular and structural imaging; **preliminary potential for morphologic characterization of MF lesions**	MF-specific evidence is based on very small studies; diagnostic accuracy and clinical applications remain unvalidated. **Very limited/emerging evidence.**
**Conventional OCT**	Vertical structural optical imaging; greater depth but limited cellular detail	Altered skin architecture, poorly defined DEJ and dermal reflectivity	Non-invasive structural assessment; potential for lesion morphology/depth characterization	Very sparse MF-specific literature. **Evidence limited to small case series/reports.**
**Other emerging techniques**	Hyperspectral/multispectral imaging and related computational approaches	Spectral, vascular and perfusion-related changes	Potential for objective lesion assessment and AI-assisted classification/monitoring	Evaluated only in preliminary studies; **clinical utility remains unvalidated.**

**Abbreviations:** MF, mycosis fungoides; FMF, folliculotropic mycosis fungoides; RCM, reflectance confocal microscopy; HFUS, high frequency ultrasound; SLEB, subepidermal low-echogenic band; LC-OCT, line-field confocal optical coherence tomography; OCT, optical coherence tomography; DEJ, Dermal-Epidermal Junction; AI, artificial intelligence.

In patients with darker skin, pigmentary structures predominate over vascular features, potentially limiting the applicability of conventional dermoscopic criteria derived from lighter phototypes. Nakamura et al. [10] evaluated MF dermoscopy in SOC patients and reported thick black lines, white rosettes, and geometric white lines in classic MF lesions. In contrast, linear vessels and spermatozoa-like vessels, which have been described in lighter-skinned patients, were not observed. The authors attributed this difference to the difficulty in recognizing vascular morphology in the presence of abundant melanin, as well as to factors that limit dermoscopic light penetration to the deeper epidermis. This observation underscores the need for greater caution when generalizing currently reported vascular patterns to more diverse patient populations and highlights the importance of phototype-specific diagnostic frameworks. Overall, the greatest strength of dermoscopy lies in its accessibility and its ability to increase clinical suspicion and guide biopsy-site selection. However, none of the reported dermoscopic findings are sufficiently validated to establish the diagnosis without histopathological confirmation.

RCM, although it penetrates to a limited depth in the skin, remains a valuable imaging technique that provides cellular-level resolution [3]. Because it allows cross-sectional evaluation in vivo, it enables a broader assessment of the epidermis than histopathology [60]. These features make it useful for both the diagnosis and monitoring of various diseases [94,95]. The most frequently detected findings in the RCM examination of MF include epidermal irregularities, epidermal lymphocytes, junctional lymphocytes, and hyporefractile papillae [60,61]. Findings may be subtle in patch lesions compared to plaque lesions [66]. The prevalence of these findings varies across studies. For instance, Pautrier microabscesses, a significant finding in MF, were identified in 20% of patients in the study by Li et al. [58], whereas this figure was 53% in the study by Mancebo et al. [60], and 74% in the study by Lange-Asschenfeldt et al. [59] Similarly, although Agero et al. [57] reported Pautrier microabscesses exclusively in plaque-stage lesions, Mancebo et al. [60] identified these structures in patch-stage lesions as well, highlighting interstudy variability. Pautrier microabscesses demonstrate excellent correlation with histopathology in studies, and T-cell receptor gene rearrangement positivity was observed to be higher in lesions exhibiting Pautrier microabscesses. In a study evaluating the role of RCM in the follow-up of MF patients, Pautrier microabscesses in combination with epidermal and junctional lymphocytes and interface dermatitis findings yielded an AUC of 0.95 in assessing disease severity. In the same study, interface dermatitis (hyporefractile papillae) was also observed in some clinically normal-appearing skin areas with RCM. This finding suggested that MF may extend beyond the clinically apparent lesional areas, with such observations potentially reflecting early or subclinical disease involvement [62].

RCM findings may also vary across MF subtypes and may provide information about them. Weakly refractile cells surrounding and infiltrating the follicular epithelium and mucin deposition are suggestive of folliculotropism [67], whereas large, bright, round, pleomorphic cells have been reported as a clue to large-cell transformation [63]. Among clinical variants, dilation of dermal blood vessels has been observed in erythematous MF, while hyperrefractive papillae at the basal layer have been described in hyperpigmented MF [60]. However, these observations are based on a limited number of cases and require confirmation in larger studies.

Although RCM provides a high-resolution image and has high specificity in differentiating MF from other erythemato-squamous diseases, its sensitivity has been found to be low. Distinguishing cell subtypes is difficult because RCM does not show nuclear and intracellular features. Additionally, lesions with significant hyperkeratosis, crusting, or ulceration can reduce the quality of highly reflective surface imaging [96]. Consequently, differentiation from other diseases is challenging [64]. It is insufficient for evaluating tumor lesions because its resolution decreases in deeper layers. Furthermore, correlation with histopathology is difficult because it provides evaluation in horizontal sections. Thus, findings such as epidermal irregularities, spongiosis, and Pautrier microabscesses may be less common or absent in histopathology, but may be present in RCM as it provides a broader perspective by presenting horizontal sections [97]. At present, RCM appears to be most applicable in tertiary referral centers with expertise in confocal microscopy, where it may complement clinical examination and histopathological assessment.

HFUS is a practical, relatively low-cost, and non-invasive imaging modality that requires less time to perform than RCM [73,74]. The most commonly used HFUS parameter for evaluating MF is the SLEB, although this finding may also be observed in inflammatory dermatoses due to inflammation and edema. In patients with MF, SLEB thickness has been shown to correlate with the degree of T-cell infiltration on histopathology. However, SLEB thickness and the histopathologic thickness of T-cell infiltration are not identical, with SLEB generally appearing thicker. This discrepancy is thought to result from tissue shrinkage during sectioning following biopsy. SLEB has also been shown to become thicker and more irregular as lesions progress from patch to plaque and tumor stages, further supporting its association with lymphocytic infiltration [75,77]. SLEB has likewise been identified as a useful parameter for treatment monitoring. In the study by Polanska et al., SLEB thickness decreased with treatment and disappeared completely in patients who achieved a complete response [72]. In contrast, Cison et al. [77] reported that although SLEB regressed following treatment, it was generally not completely eliminated, with less pronounced reduction observed particularly in younger patients. Accordingly, the authors suggested that closer follow-up may be warranted in cases where SLEB does not fully resolve. The value of non-invasive imaging for objective treatment assessment has also been recognized in other dermatologic indications involving light-based therapies [98]. Another advantage of HFUS is its potential to provide information on MF subtypes. In the study of Wang et al. [75], hypoechoic areas around hair follicles were observed in patients with folliculotropism. While HFUS has its advantages, the role of HFUS in differential diagnosis remains limited because SLEB is not specific to MF. In one study, a thinner SLEB was reported to favor MF over psoriasis and atopic dermatitis when differentiating early-stage MF from inflammatory dermatoses [78]; however, this finding was not confirmed in the subsequent studies [20]. Taken together, the available evidence suggests that HFUS may currently have greater value in monitoring disease activity than in establishing the diagnosis of MF.

## 5. Limitations

The primary limitation in this area was the small sample size, as most studies were limited to case reports, case series, or small cohort studies. Furthermore, significant clinical heterogeneity—including diverse clinical subtypes and disease stages—further restricted the generalizability of the findings by dividing the already limited patient population into even smaller subgroups. Variability in image acquisition methods—including differences in dermoscopic magnification, HFUS examination frequency, and instrument calibration—also contributed to inconsistencies in the reported findings. Clinical overlap between MF and numerous benign dermatological conditions further exacerbated these inconsistencies. The current body of evidence may also be influenced by publication bias, as studies reporting distinctive features are more likely to be published than those with negative findings. Consequently, these findings should be interpreted with caution from a diagnostic standpoint. Furthermore, the lack of standardized criteria for evaluation increased inter-operator variability, preventing direct comparisons between studies. Therefore, to improve diagnostic accuracy, it will be necessary to establish a universal consensus on descriptors in imaging techniques and adopt a systematic framework to standardize terminology.

## 6. Future Perspectives

To overcome the main limitations of earlier studies, such as small sample sizes and publication bias, future research should involve multicenter studies with standardized imaging protocols. These approaches would enhance the generalizability and reproducibility of imaging findings. It is also important to compare different imaging methods directly in the same group of patients. Doing so will help clarify the strengths, weaknesses, clinical uses, and optimal patient selection criteria for each imaging technique.

In the field of non-invasive skin imaging, AI has recently contributed to increasing success rates in diagnosis and differential diagnosis. AI has been found to be more successful than clinicians in diagnosing melanoma and non-melanoma skin cancers using dermoscopic images [99]. Studies are also being conducted on the use of AI in the diagnosis of these same diseases with confocal microscopy, and successful results are being obtained [100]. While there are not yet enough studies on this topic in skin imaging of MF, AI algorithms can significantly improve differential diagnosis and diagnostic success rates.

Furthermore, a recent study used a fluorescent contrast agent called PARPi-FL in the detection of basal cell carcinoma with confocal microscopy, visualizing BCC up to a depth of ~100 µm in ex vivo human tissue and in vivo mouse models [101]. These significant developments can improve the success of superficial imaging techniques and minimize the need for biopsy for diagnosis. These advancements can also prevent delays in the diagnosis of MF, a type of cutaneous T-cell lymphoma.

The establishment of standardized imaging criteria for mycosis fungoides constitutes an additional research priority. Similar to consensus-based dermoscopic algorithms for melanoma, validated diagnostic criteria for MF could improve biopsy-site selection, enable earlier diagnosis, and support more consistent interpretation of imaging results. However, these criteria must be validated in large, prospective multicenter studies prior to their integration into routine clinical practice.

## 7. Conclusions

While skin imaging techniques are valuable for diagnosing mycosis fungoides, histopathological examination remains the gold standard. Nevertheless, imaging modalities enable earlier detection and help minimize unnecessary biopsies by guiding the selection of optimal biopsy sites. They also aid in monitoring treatment response and detecting residual disease. Given these advantages, non-invasive imaging techniques may play an important complementary role in the management of MF, and further research in this field is warranted.

## Figures and Tables

**Table 1:** Search Strategy.

**Pubmed** ((“high-frequency ultrasound”[tiab] OR “high frequency ultrasound”[tiab] OR HFUS[tiab] OR “ultra-high-frequency ultrasound”[tiab] OR “ultra high frequency ultrasound”[tiab]) OR (“reflectance confocal microscopy”[Mesh] OR “reflectance confocal microscopy”[tiab] OR RCM[tiab]) OR (“dermoscopy”[Mesh] OR dermoscopy[tiab] OR dermatoscopy[tiab]) OR (“optical coherence tomography”[Mesh] OR “optical coherence tomography”[tiab] OR OCT[tiab] OR “line-field optical coherence tomography”[tiab] OR LC-OCT[tiab]) OR (trichoscopy[tiab]) OR (“non-invasive skin imaging”[tiab] OR “non-invasive skin imaging”[tiab] OR “non-invasive imaging”[tiab] OR “non-invasive imaging”[tiab])) AND (“Mycosis Fungoides”[Mesh] OR “mycosis fungoides”[tiab] OR “Lymphoma, T-Cell, Cutaneous”[Mesh] OR “cutaneous T-cell lymphoma”[tiab] OR CTCL[tiab])
**EMBASE** (‘high frequency ultrasound’/exp OR ‘ultra high frequency ultrasound’:ab,ti OR hfus:ab,ti OR ‘reflectance confocal microscopy’/exp OR ‘reflectance confocal microscopy’:ab,ti OR rcm:ab,ti OR ‘dermoscopy’/exp OR dermoscopy:ab,ti OR dermatoscopy:ab,ti OR ‘optical coherence tomography’/exp OR ‘optical coherence tomography’:ab,ti OR oct:ab,ti OR ‘line field optical coherence tomography’:ab,ti OR ‘lc-oct’:ab,ti OR trichoscopy:ab,ti OR ‘non-invasive skin imaging’:ab,ti OR ‘non-invasive skin imaging’:ab,ti OR ‘non-invasive dermatologic imaging’:ab,ti OR ‘non-invasive dermatologic imaging’:ab,ti) AND (‘mycosis fungoides’/exp OR ‘mycosis fungoides’:ab,ti OR ‘cutaneous t-cell lymphoma’/exp OR ‘cutaneous t-cell lymphoma’:ab,ti OR ctcl:ab,ti OR ‘cutaneous lymphoma’/exp OR ‘cutaneous lymphoma’:ab,ti) AND [article]/lim AND [humans]/lim AND [english]/lim

## Data Availability

No new data were created or analyzed in this study. Data sharing is not applicable to this article.
